# FACI is a novel clathrin adaptor protein 2-binding protein that facilitates low-density lipoprotein endocytosis

**DOI:** 10.1186/s13578-023-01023-5

**Published:** 2023-04-18

**Authors:** Yun Cheng, Xiao-Zhuo Kang, Pearl Chan, Pak-Hin Hinson Cheung, Tao Cheng, Zi-wei Ye, Chi-Ping Chan, Cheng-Han Yu, Dong-Yan Jin

**Affiliations:** 1 School of Biomedical Sciences, The University of Hong Kong, 21 Sassoon Road, Pokfulam, Hong Kong; 2 State Key Laboratory of Liver Research, The University of Hong Kong, 21 Sassoon Road, Pokfulam, Hong Kong

**Keywords:** FACI, Cholesterol uptake, Low-density lipoprotein receptor, Clathrin adaptors, Endocytosis, Hypercholesterolemia

## Abstract

**Background:**

Cholesterol plays a vital role in multiple physiological processes. Cellular uptake of cholesterol is mediated primarily through endocytosis of low-density lipoprotein (LDL) receptor. New modifiers of this process remain to be characterized. Particularly, the role of fasting- and CREB-H-induced (FACI) protein in cholesterol homeostasis merits further investigation.

**Methods:**

Interactome profiling by proximity labeling and affinity purification − mass spectrometry was performed. Total internal reflection fluorescence microscopy and confocal immunofluorescence microscopy were used to analyze protein co-localization and interaction. Mutational analysis was carried out to define the domain and residues required for FACI localization and function. Endocytosis was traced by fluorescent cargos. LDL uptake in cultured cells and diet-induced hypercholesterolemia in mice were assessed.

**Results:**

FACI interacted with proteins critically involved in clathrin-mediated endocytosis, vesicle trafficking, and membrane cytoskeleton. FACI localized to clathrin-coated pits (CCP) on plasma membranes. FACI contains a conserved DxxxLI motif, which mediates its binding with the adaptor protein 2 (AP2) complex. Disruption of this motif of FACI abolished its CCP localization but didn’t affect its association with plasma membrane. Cholesterol was found to facilitate FACI transport from plasma membrane to endocytic recycling compartment in a clathrin- and cytoskeleton-dependent manner. LDL endocytosis was enhanced in FACI-overexpressed AML12 cells but impaired in FACI-depleted HeLa cells. In vivo study indicated that hepatic FACI overexpression alleviated diet-induced hypercholesterolemia in mice.

**Conclusions:**

FACI facilitates LDL endocytosis through its interaction with the AP2 complex.

**Supplementary Information:**

The online version contains supplementary material available at 10.1186/s13578-023-01023-5.

## Introduction

Endocytosis is a cellular process in which cargo molecules are internalized from exterior to interior. It is pivotal to nutrient internalization, signal transduction and the composition of the plasma membrane (PM). Multiple types of endocytosis have been identified in eukaryotic cells, among which clathrin-mediated endocytosis (CME) is the most important [1]. CME starts from the recruitment of endocytic adaptors from the cytoplasm by PM phosphatidylinositol 4,5-bisphosphate (PIP2). Endocytic adaptors further recruit cargos, clathrin and endocytic accessory proteins to form clathrin-coated pits (CCPs). CCPs were invaginated with the help of endocytic accessory proteins and scissored from the PM by dynamin. In cytoplasm, the endocytic machinery is disassembled from clathrin-coated vesicles (CCVs), and the nascent uncoated vesicles are fused with early endosomes (EE) for cargo sorting [2, 3].

Endocytic adaptors link cargos into the CCPs. Clathrin adaptor protein 2 (AP2) complex is one of the most crucial endocytic adaptors for CME. The AP2 complex is a heterotetramer consisting of four subunits: α, β2, μ2 and σ2. AP2 selects cargos by directly binding to the sorting signals of cargo proteins. YxxΦ and [DE]xxxL[LI], where x can be any residue and Φ is a hydrophobic amino acid, are the two most common motifs for AP2 recognition [4]. Other endocytic adaptors include endocytic adaptor disabled homolog 2 (Dab2), autosomal recessive hypercholesterolemia (ARH) and epsins, which recognize and sort specific cargo into CCPs [5, 6].

In humans, cholesterols are derived from two sources: endogenous biosynthesis and exogenous dietary uptake. The small intestine is in charge of exogenous cholesterol uptake via Niemann-Pick C1-like 1 (NPC1L1) protein. The liver and intestine are primarily responsible for endogenous cholesterol biosynthesis [7]. The absorbed and synthesized cholesterols, together with triglycerides, are packaged into chylomicrons (CMs) in the intestine or very-low-density lipoproteins (VLDLs) in the liver and released into the circulation for utilization by other organs. After the removal of triglycerides by peripheral tissues, CMs and VLDLs are enriched in cholesterol and converted into CM remnants and low-density lipoproteins (LDLs). LDLs exist in higher levels and have longer half-lives than CM remnants. LDLs are therefore the major lipoproteins delivering cholesterol to peripheral tissues [8, 9]. Increased LDL-cholesterol (LDL-C) level is strongly associated with the risk of atherosclerosis, a leading cause of cardiovascular death worldwide. Most LDLs are ultimately cleared by the liver. Intestine may also clear LDLs through the transintestinal cholesterol excretion (TICE) pathway [10].

LDL uptake is mediated via the CME of the LDL receptor (LDLR). LDL attachment to the extracellular domain of LDLR leads to conformational change of LDLR. The cytoplasmic NPxY motif of LDLR is exposed and recognized by Dab2 and ARH. Dab2 and ARH further recruit clathrin and other components of the endocytic machinery to form CCVs, leading to the internalization of the LDL-LDLR complex [9, 11]. AP2 is required for ARH-mediated LDLR endocytosis, however, it is seemingly dispensable for Dab2 function [12]. Internalized LDL-LDLR complex is delivered into the EE, where the acidic environment leads to their dissociation. LDL particles are sorted to the late endosome (LE) and eventually to the lysosome for further processing, while LDLRs are recycled back to the PM through the CCC complex-dependent pathway [11, 13]. The endocytosed cholesterol is mainly stored in the PM and endocytic recycling compartment (ERC). Some cholesterol is converted into cholesterol ester reserved in lipid droplets [14]. Proprotein convertase subtilisin/kexin type 9 (PCSK9) binds to the LDLR at the cell surface and redirects it into lysosome for degradation [15, 16]. E3 ubiquitin ligase inducible degrader of the LDL receptor (IDOL) catalyzes LDLR ubiquitylation and mediates its degradation [17].cAMP-responsive element-binding protein H (CREB-H) is a liver- and intestine-enriched transcription factor, which plays an important role in lipid metabolism [18, 19]. CREB-H knockout (CREB-H^−/−^) mice showed dyslipidemia and impaired lipoprotein clearance by livers [20, 21]. We previously identified fasting- and CREB-H-induced (FACI) gene, also known as C11ORF86 or FLJ22675, to be a novel target gene of CREB-H [22]. FACI is a small protein with around 115 amino acids and is abundantly expressed in the liver and small intestine. FACI localizes to the PM and ERC by its phosphoinositide-binding motif. Under high-fat diet, *Faci*^−/−^ mice showed higher plasma cholesterol than wild type (WT) mice [22]. We speculate that the cholesterol disorder of *Faci*^−/−^ mice might not solely be caused by diet-induced obesity. FACI deficiency may directly impair cholesterol homeostasis. In this study, we characterized the interactome of FACI. We found that FACI localizes to CCPs and directly binds AP2 through its DxxxLI motif. FACI facilitated LDL uptake. Hepatic FACI overexpression alleviated diet-induced hypercholesterolemia in mice. Our study revealed one regulatory function of FACI in cholesterol metabolism.

## Materials and methods

### Plasmids, reagents, and oligonucleotides

Plasmids, reagents, primers and sgRNA oligonucleotides for FACI gene knockout have been listed in Additional file 1: Table S1.

We cloned FACI open reading frame (ORF) into various vectors including mRuby2-C1, mEmerald-C1, pCW57-GFP-2A-MCS and pCDH-Myc-BioID2. The mRuby2-C1 and mEmerald-C1 were gifts from Michael Davidson [23]. The pCW57-GFP-2A-MCS is an all-in-one doxycycline inducible lentiviral vector gifted by Adam Karpf [24]. The pCDH-Myc-BioID2 is a lentiviral vector designed to stably express BioID2-tagged bait proteins. The pCDH-Myc-BioID2 was generated by us based on myc-BioID2-MCS and pCDH-CMV-MCS-EF1-Puro (System Biosciences). Plasmid myc-BioID2-MCS was a gift from Kyle Roux [25]. We also subcloned FACI ORF into AAV expression vector system, which was kindly provided by Sharon C Cunningham and Ian E Alexander [26].

Plasmids CLC-pmCherryC1 and AP2u2-mCherry were gifts from Christien Merrifield [27]. CAV1-mCherry was provided by Ari Helenius [28]. Plasmid mCherry-Dab2 has been previously described [29, 30]. Plasmid mCherry-Rab11a-7 was a gift from Michael Davidson. Plasmid α‐eGFP was a gift from Sandra Schmid [31]. GFP-LDLR was a gift from Gary Banker and Marvin Bentley [32]. LentiCRISPRv2 was provided by Feng Zhang [33].

### Cell culture and transfection

Human embryonic kidney cell line HEK293T and human cervical adenocarcinoma cell line HeLa were cultured in Dulbecco’s Modified Eagle’s Medium (DMEM, ATCC) containing 10% fetal bovine serum (FBS, Life Technologies). Mouse immortal hepatic cell line AML12 was cultured in DMEM/F-12 medium (Gibco) supplemented with insulin, transferrin, selenium (ITS; Gibco), 40 ng/mL dexamethasone (Sigma) and 10% FBS. Human colorectal adenocarcinoma cell line Caco-2 and human hepatic cell line HepG2 were cultured in Eagle's Minimum Essential Medium (ATCC) containing 10% FBS. All cell lines were maintained at 37 °C with a humidified atmosphere containing 5% CO_2_.

HEK293T and HeLa cells were transfected via GeneJuice transfection reagent (Novagen). AML12 and Caco-2 cells were transfected using Lipofectamine 3000 (Invitrogen) with P3000 (Invitrogen). Transfection procedures were performed as instructed by manufacturers.

### RNA extraction and real time RT-PCR

RNAiso Plus reagent (TaKaRa) was used to extract total RNA in accordance with the manufacturer’s instructions. The extracted RNA was incubated with DNase I (Ambion) at 37 ℃ for 30 min to digest the remaining genomic DNA. Reverse transcription was performed using the Transcriptor First Strand cDNA Synthesis reagents (Roche).

TB green PCR master mix was purchased from Takara and Real-time quantitative PCR was conducted by StepOne™ real-time PCR system (Applied Biosystems). Gene expression was normalized to Actb expression by 2-(ΔΔCt) method. Primer sequences used are listed in Additional file 1: Table S1.

### Stable cell line generation

A total of 5 stable cell lines (AML12-BioID2, AML12-BioID2-FACI, AML12-V5-FACI, AML12-CTR and AML12-mRuby2-FACI) were generated with the lentiviral vector system in this paper (listed in Additional file 1: Table S1) using the previously described protocol [34]. Briefly, the lentiviral backbone plasmid was co-transfected into HEK293T cells with package plasmids. After 48-h incubation, the culture medium was collected and filtered through a 0.22-µm filter. Virus-containing supernatant was further concentrated with Lenti-X™ concentrator (Takara) and added to pre-seeded target cells with 8 μg/ml polybrene. The transduction was allowed to continue for 48 h, followed by puromycin selection to kill the lentivirus-negative cells. Drug selection lasted for 2 weeks until a stable cell line was obtained.

FACI^−/−^ HeLa cell lines were generated by CRISPR/Cas9 technique. Two gRNAs targeting the sequence closest to ATG codon of FACI were designed (crispr.mit.edu) and subcloned into lentiCRISPRv2 plasmids. The two gRNA plasmids as well as lentiCRISPRv2 as a control were transfected into HeLa cells. After 3-day selection with puromycin (4 μg/ml), single colonies were picked and expanded. The gRNA-targeted region of each colony was amplified with KAPA high-fidelity DNA polymerase and cloned into pGEM-T vectors. For each HeLa colony, six TA-cloning products were sequenced. According to the sequencing results, two FACI^−/−^ and one control HeLa colonies were selected for further assay. Primers for TA-cloning and sgRNA oligonucleotides were listed in Additional file 1: Table S1. The stable cells were then cultured in normal culture medium supplemented with 2 µg/mL puromycin.

### Protein extraction and immunoblotting

Immunoblotting was performed as described previously [35]. Cells were lysed in NP40 lysis buffer (150 mM NaCl, 1.0% NP-40 and 25 mM Tris–HCl, pH 7.4) supplemented with protease inhibitor cocktails (Roche) by 30 min incubation at 4 °C. Protein concentration of lysates was measured with Bradford dye-binding method (BioRad). Protein samples were separated by SDS-PAGE, electroblotted onto polyvinylidene difluoride membranes (Millipore), incubated with primary and secondary antibodies sequentially and visualized by enhanced chemiluminescence (Amersham).

### Proximity-dependent biotin identification (BioID)

BioID assays were performed in AML12 cells, according to the previously described protocol [36]. Two AML12 stable cell lines (i.e., AML12-BioID2 and AML12-BioID2-FACI) were generated. Cells were cultured in four 15 cm culture dishes. When cells reached approximately 80% confluency, fresh medium with 50 μM biotin (Thermo Fisher) was replenished for another 24 h. Cells were lysed in the lysis buffer (8 M urea in 50 mM Tris–HCl, pH 7.4, 1 × protease inhibitor Cocktail, 1 mM dithiothreitol and 1% Triton X-100) at 4 °C. Cell lysates were then sonicated and digested with Benzonase nuclease (Merck). Protein supernatants were obtained by centrifuging the crude lysate for 16,500 × g for 20 min. Protein supernatants were mixed with equilibrated streptavidin Dynabeads (Invitrogen), followed by overnight incubation on a rotator at 4 °C. Next day, the beads were washed four times with wash buffer (50 mM Tris–HCl, pH 7.4, 8 M urea and 1 × protease inhibitor Cocktail). The supernatant was then removed by centrifugation and the remaining beads were boiled with SDS-PAGE sample loading buffer. The boiled samples were resolved by SDS-PAGE and examined by immunoblotting.

### Immunoprecipitation (IP)

Immunoprecipitation was carried out as described [37]. Cells were collected and lysed in the NP-40 lysis buffer supplemented with protease inhibitor cocktails. Cell lysates were mixed with antibodies at 4 °C overnight followed by the addition of protein G Dynabeads (Invitrogen) for 2 h at 4 °C. The immunoprecipitates were collected by centrifugation, washed three times with wash buffer, and eluted with the 2 × SDS-PAGE sample loading buffer by boiling. The immunoprecipitates were separated by SDS-PAGE and analyzed by immunoblotting.

For IP-MS assay, AML12-CTR and AML12-V5-FACI stable cell lines were used. Cells were cultured on four 15 cm culture dishes until reaching 80% confluence. Doxycycline was then supplemented into cell media for 24 h to induce V5-FACI expression. Cells were lysed and immunoprecipitated by anti-V5 antibody (Invitrogen). Immunoprecipitated samples were examined by immunoblotting and silver staining.

### Protein identification by liquid chromatography with tandem mass spectrometry (LC–MS/MS)

Two independent BioID assays (BioID-R1 and -R2) and two independent IP-MS assays (IP-R1 and -R2) were conducted in AML12 cells. BioID and IP samples were stacked by SDS-PAGE and visualized by Coomassie blue staining. The stained gel lanes were cut into slices and sent to the Proteomics and Metabolomics Core Facility of the University of Hong Kong for LC–MS/MS analysis. The methods for LC–MS/MS have been described previously [38]. Briefly, gel slices were subjected to reduction and alkylation by 10 mM TCEP and 55 mM 2-chloroacetamide, respectively. Proteins were digested with trypsin (1 ng/μl) overnight at 37 °C. Tryptic peptides were extracted, pooled, dried, and desalted sequentially. Eluted peptides were analyzed with UltiMate 3000 RSLCnano UHPLC coupled to Orbitrap Fusion Tribrid Lumos mass spectrometer (Thermo Fisher) or nanoelute UHPLC coupled to timsTOF PRO mass spectrometer (Bruker).

Raw mass spectrometric data were searched against the mouse UniProt FASTA database with MaxQuant software. Label-free methods were used for protein quantitation. The cutoffs for potential “FACI interactors” are iBAQ intensity of greater than 2^1.5^-fold (~ 2.8 fold), protein score of greater than 35, and MSMS counts of greater than 2 relative to the control. “Protein score” calculated by Maxquant is derived from peptide posterior error probabilities. The detailed information of potential FACI-interactors was listed in Additional file 2: Table S2 and Additional file 3: Table S3.

High-confidence “FACI-interacting proteins” were selected from the potential FACI-interactors which were identified more than twice in BioID and IP-MS assays. Protein–protein interaction networks were analyzed by STRING and visualized by Cytoscape. GO analysis was by DAVID and visualized by R-studio. The selected high-confidence FACI-interacting proteins were listed in Additional file 4: Table S4.

### Site-directed mutagenesis

The mEmerald-FACI plasmid was mutated by Q5 Site-Directed Mutagenesis Kit (New England Biolabs). Mutation primers were designed by online software NEBaseChanger. Mutagenesis PCR, kinase-ligase-DpnI (KLD) treatment and transformation were conducted following the manufacturer’s instructions. All the mutations had been confirmed by DNA sequencing (BGI, Shenzhen).

### Total internal reflection fluorescence microscopy (TIRFM)

TIRFM was performed with an inverted microscope (Nikon Eclipse Ti2-E) with TIRFM module using a 100 × 1.49 oil DIC objective. Cells were seeded in a 20 mm confocal dish (Mattek) and transfected with target plasmids for 1 ~ 2 days. mEmerald- and mCherry-labeled proteins were visualized sequentially by 488 nm and 561 nm laser. For live cell imaging, cells were cultured in pre-warmed phenol red-free DMEM medium (Life Technologies). The image acquisition was set for 8 min with 2-s interval.

### Spinning disc confocal microscopy (SDCM)

SDCM was performed with PE UltraVIEW VoX spinning disk confocal microscope (Perkin Elmer) using a 100 × 1.45 oil objective. Cells were seeded in a 35 mm glass bottom confocal dish with 14 mm Imaging Windows (Mattek) and transfected with target plasmids for 1 day. EGFP-labeled proteins and TopFluor cholesterol (Avanti Polar Lipids) were visualized by a 488 nm laser, and mRuby2-labeled proteins were visualized by a 561 nm laser. For z-stack confocal scan, the axial increment per optical slice was set as 0.479 µm. For live cell imaging, cells were cultured in pre-warmed phenol red-free DMEM medium (Life Technologies) and kept on the TOKAI HIT stage-top incubator module (37 °C and 5% CO_2_) on the microscope. The image acquisition was set for 10 min within 2 ~ 10 s intervals.

### Drug treatment

AML12-mRuby2-FACI cells were cultured in doxycycline-containing media for one day to induce the expression of mRuby2-FACI. Before the drug treatment, the culture medium was replaced with fresh medium without doxycycline (DMEM/F-12 medium supplemented with 10% FBS).

For cholesterol uptake assay, AML12-mRuby2-FACI cells were treated with 40 µg/mL cholesterol-water soluble (Sigma) for 1 h. TopFluor cholesterol (2 ng/mL) was added together with water soluble-cholesterol if necessary.

For CME inhibition and following cholesterol uptake assay, AML12-mRuby2-FACI cells were pretreated with 30 µM Pitstop-2 (Abcam) for 15 min, then water soluble-cholesterol (Sigma, 40 µg/mL) was added into the culture media with 2 ng/mL TopFluor cholesterol for 1 h. Cells with mock treatment were used as control. For cholesterol depletion assay, AML12-mRuby2-FACI cells were treated with cholesterol-depleting medium for 2 h. Cholesterol-depleting medium is the DMEM/F-12 medium supplemented with 10% lipoprotein-deficient serum (LPDS), 1 μM lovastatin and 5 mM MβCD.

For cytoskeleton disruption and following cholesterol uptake assay, AML12-mRuby2-FACI cells were incubated with 2 µM CytD or 2 µM NDZ for 30 min, water- soluble cholesterol (Sigma) was then added into the culture media to 40 µg/mL for 1 h. Cells with mock cholesterol treatment were used as control. In the mock cholesterol uptake treatment, the same volume of PBS rather than cholesterol-water soluble was added after the drug pretreatment.

### Immunofluorescent staining

Cells were fixed with 4% paraformaldehyde for 15 min, permeabilized for 4 min with 0.1% Triton in PBS, blocked with 3% BSA for 1 h, and incubated with primary antibody overnight at 4 °C sequentially. After washing, the cells were incubated with a secondary antibody conjugated with fluorophore (1:400) in a dark place at room temperature for 1 h. The cells were further rinsed and then mounted with DAPI-containing mounting media. Confocal images were acquired with the confocal laser scanning microscopes LSM780 and LSM880 (Zeiss).

### LDL, transferrin and EGF uptake assays, and flow cytometry

LDL, transferrin and EGF uptake assays using pHrodo™ red-LDL (Thermofisher), pHrodo™ red transferrin conjugate (Thermo Fisher) and pHrodo™ red EGF conjugate (Thermo Fisher) were carried out following manufacturer’s protocols. HeLa cells and AML12 cells were seeded in 12-well plates and fasted for the instructed time before the uptake assay. Cells were then incubated with medium containing pHrodo-red labeled LDL (20 µg/mL), transferrin (25 µg/mL) and EGF (2 µg/mL) at 37 °C for 20 min to 2 h.

The cells of interest were then washed with ice-cold PBS, digested, and harvested by centrifugation at 300 × g for 3 min. The cells were directly resuspended in the ice-cold FACS buffer (PBS with 2 mM EDTA and 0.5% BSA, pH 7.4) and the fluorescent signals were detected using NovoCyte Quanteon flow cytometer. The acquired data were analyzed with FlowJo.

### AAV-mediated transgene expression in mice

The recombinant AAV2/8 system was used for in vivo liver-directed transgene expression. Two recombinant AAVs (rAAVs) were used in this study: a FACI-expressing rAAV (AAV-FACI) and an EGFP-expressing rAVV (AAV-GFP) as control. Preparation and purification of rAAV have been described previously [26]. For viral delivery, mice were intraperitoneally injected with rAAV with a dose of 1 × 10^11^ genome copy/mouse.

### Mice

The 6-week-old C57BL/6 mice (male) were randomly selected and infected with AAV-GFP (n = 7) and AAV-FACI (n = 8). After two weeks of infection, mice were fed with a high-cholesterol diet (D12108C, Research Diet) for another month. The 8-week-old C57BL/6 mice (male, n = 6) and FACI^−/−^ mice (male, n = 8) were randomly selected and fed with the high-cholesterol diet (Research Diet D12108C) for one month. FACI^−/−^ mice were generated by Cyagen (Santa Clara, CA) [22]. Mice were anesthetized, and the blood and liver tissues were collected for further assays. Experimental mice were housed in the Center for Comparative Medicine Research of the University of Hong Kong with a daylight cycle from 7 am to 7 pm. All animal experiments were approved by the Committee on the Use of Live Animals in Teaching and Research at the University of Hong Kong (Animal Ethics Approval No. 3777–15 and 4776–18).

### Blood biochemistry tests

All blood biochemistry tests were conducted following manufacturer’s instructions. Total triglyceride (TG) and total cholesterol (TC) were examined by kits from Stanbio. LDL-C and HDL-C were examined by kits from JianCheng Biotech (Nanjing, China).

### Lipid extraction

Mice were anesthetized, and liver tissues were collected for lipid extraction following the Bligh and Dyer method [39]. Mouse liver tissues (50 mg) were homogenized in 760 μl cold PBS and mixed with 3 ml of chloroform/methanol (1:2, v/v) solution. After vortex, 1 ml chloroform and 1 ml 0.88% NaCl solution was further added and mixed. The mixtures were centrifuged at 2000 × g for 15 min at 4 °C. The organic phases were dried and reconstituted in 2% Triton X-100 in H_2_O. Liver TG and cholesterol levels were determined by the commercial kits (Stanbio) and normalized to liver tissue weights.

### Statistics

All experiments were performed with at least three independent biological replicates. Values are the means of biological replicates ± standard deviation (SD). GraphPad Prism 8 was used for statistical analysis. Statistical analyses were performed with two-tailed unpaired Student’s t test or one-way ANOVA followed by Tukey post hoc comparison.

## Results

### Interactome analysis suggests an endocytic function of FACI

**Fig. 1:**
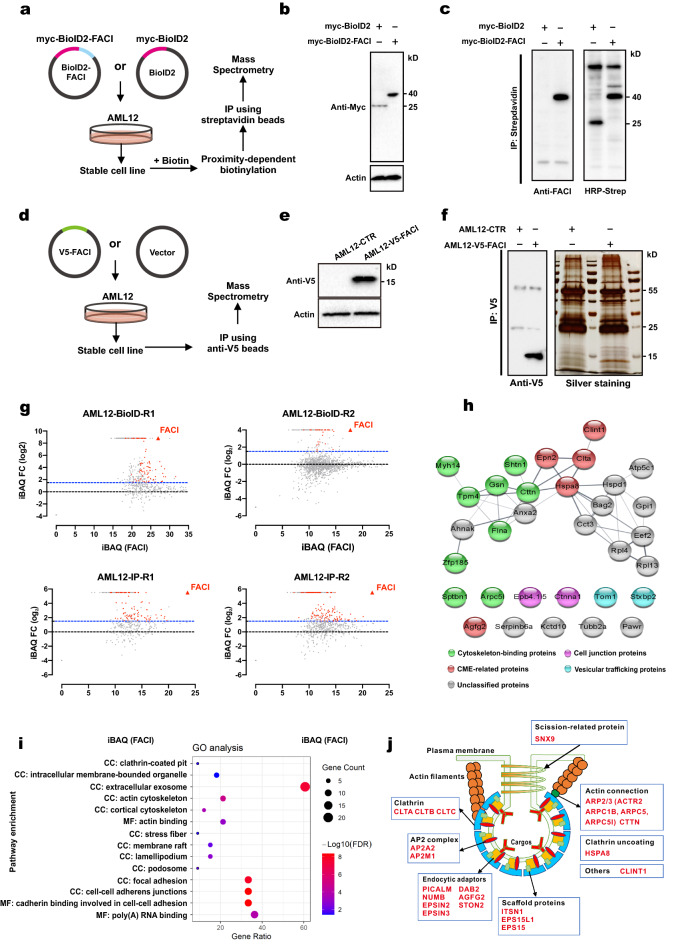
Proteomic analysis of FACI-interacting proteins by BioID and IP-MS. **A** Schematic diagram of the BioID workflow. Myc-BioID2-FACI fusion protein was used as the bait. **B** AML12 cell lines stably expressing myc-BioID2-FACI (AML12-BioD2-FACI) and myc-BioID2 (AML12-BioD2) were generated and verified by immunoblotting. **C** Immunoprecipitation (IP). Biotin-labeled proteins from AML12-BioID2 and AML12-BioID2-FACI cells were captured by streptavidin and separated by SDS-PAGE. Samples were probed with anti-FACI (left) and HRP-streptavidin (HRP-Strep; right). **D** Schematic diagram of the IP-MS workflow. V5-tagged FACI protein was used as the bait. **E** AML12 cell lines stably expressing V5-FACI (AML12-V5-FACI) were generated and verified by immunoblotting. A mock AML12 stable cell line (AML12-CTR) was generated and used as a control. **F** Immunoprecipitation (IP). Protein lysates from AML12-CTR and AML12-V5-FACI cells were incubated with anti-V5 antibody. Immunoprecipitated proteins were separated by SDS-PAGE, followed by immunoblotting (left) and silver staining (right). **G** Volcano plots illustrating two independent BioID assays (BioID-R1 and -R2) and two independent IP-MS assays (IP-R1 and -R2) in AML12 cells. Y-axis represents log_2_ fold change (FC) of protein iBAQ intensity of the FACI-immunoprecipitated group relative to the control group. X-axis indicates protein iBAQ values of the FACI-immunoprecipitated group. FACI-immunoprecipitated group refers to AML12-V5-FACI or AML12-BioID2-FACI. Control group refers to AML12-CTR or AML12-BioID2. Proteins with iBAQ value larger than 2^1.5^-fold, protein score higher than 30 and MSMS counts of greater than 2 relative to the control were recognized as “potential FACI interactors” and highlighted in red. The detailed information of “potential FACI interactors” was listed in Additional file 2: Table S2 and Additional file 3: Table S3. iBAQ: intensity-based absolute quantification. **H** STRING protein–protein interaction networks of FACI-interacting proteins. The 32 high-confidence FACI-interacting proteins were selected from the potential FACI-interactors which were identified more than twice in BioID and IP-MS assays. Network nodes were differentially colored based on protein functions. Detailed information of the 32 FACI-interacting proteins were listed in Additional file 4: Table S4. **I** The bubble plot depicting gene ontology (GO) of FACI-interacting proteins. Y-axis represents the GO terms. X-axis indicates gene ratio. Bubble colors represent -log_10_(FDR) and bubble sizes indicate the number of genes. **J** Schematic diagram of “potential FACI interactors” involved in CCPs

The interactome of FACI was investigated by the proximity-dependent biotin identification (BioID) technique and by immunoprecipitation-mass spectrometry (IP-MS). For BioID, AML12 cell lines stably expressing myc-BioID2-FACI (AML12-BioID2-FACI) and myc-BioID2 (AML12-BioID2) were generated. After 24-h treatment with biotin, the stable cells were lysed, and biotinylated proteins were pulled down with streptavidin beads (Fig. 1A). Constitutive expression of myc-BioID2-FACI of 40 kD and myc-BioID2 of 26 kD in the respective AML12 cell lines was verified by immunoblotting (Fig. 1B). The streptavidin–horseradish peroxidase (HRP) immunoblot assays revealed successful biotinylation of myc-BioID2-FACI and other proteins in AML12-BioID2-FACI cells (Fig. 1C). For IP-MS, AML12 cell line stably expressing V5-FACI (AML12-V5-FACI) and control AML12 stable cell line stably carrying an empty vector (AML12-CTR) were constructed (Fig. 1D). Immunoblotting results indicated stable expression of V5-FACI in AML12-V5-FACI cells (Fig. 1E). Immunostaining confirmed localization of V5-FACI to the PM and ERC (Additional file 5: Fig. S1), which was consistent with previous results [22]. Total proteins of AML12-V5-FACI and AML12-CTR were harvested and immunoprecipitated with anti-V5 antibody. Silver staining and immunoprecipitation results indicated successful enrichment of V5-FACI of 14 kD (Fig. 1F). Immunoprecipitated samples from BioID and IP were digested and analyzed by liquid chromatography-tandem mass spectrometry (LC–MS/MS) technique.

Both BioID and IP-MS experiments were independently performed twice, which was indicated in Fig. 1G as AML12-BioID-R1 and AML12-BioID-R2 (two runs of BioID assays), as well as AML12-IP-R1 and AML12-IP-R2 (two runs of IP-MS). Label-free quantitation was used for mass spectrometric analysis. Proteins with intensity based absolute quantitation (iBAQ) value of greater than 2^1.5^-fold (~ 2.8 fold), protein score of greater than 35, and MSMS counts of greater than 2 relative to the control were selected as “potential FACI interactors”. The iBAQ intensity and other information of “potential FACI interactors” were listed in Additional file 2: Table S2 and Additional file 3: Table S3. Results of BioID and IP-MS assays were presented as the volcano plots in Fig. 1G. FACI protein was the most significantly enriched one in all four experiments, with iBAQ intensities of 2^26.95^, 2^17.73^, 2^23.57^ and 2^21.84^ in AML12-BioID-R1, AML12-BioID-R2, AML12-IP-R1 and AML12-IP-R2, respectively.

A total of 32 “potential FACI interactors” were identified at least twice by mass spectrometric analysis, and further selected as high-confidence “FACI interactors” (Additional file 4: Table S4). The protein–protein interaction networks of the 32 proteins were constructed with STRING (Fig. 1H) [40]. Among the 32 proteins, there were 5 CME-related proteins (CLINT1, EPN2, CLTA, HSPA8 and AGFG2), 9 cytoskeleton-binding proteins (MYH14, SHTN1, TPM4, GSN, CTTN, FLNA, ZFP185, SPTBN1 and ARPC5L), 2 vesicular trafficking proteins (TOM1 and STXBP2), 2 cell junction proteins (EPB41L5 and CTNNA1) and 14 unclassified proteins. In keeping with STRING analysis, Gene Ontology (GO) enrichment analysis of the 32 proteins also yielded similar results (Fig. 1I and Additional file 4: Table S4).

Interactome analysis of FACI suggested that FACI might be closely related to the following pathways: clathrin-coated pits, cytoskeleton, and intracellular trafficking. Particularly, many CME-related proteins were identified as potential or high-confidence FACI interactors (Fig. 1H, J), indicating strong association of FACI with CME.

### Colocalization of FACI with CME machinery

**Fig. 2:**
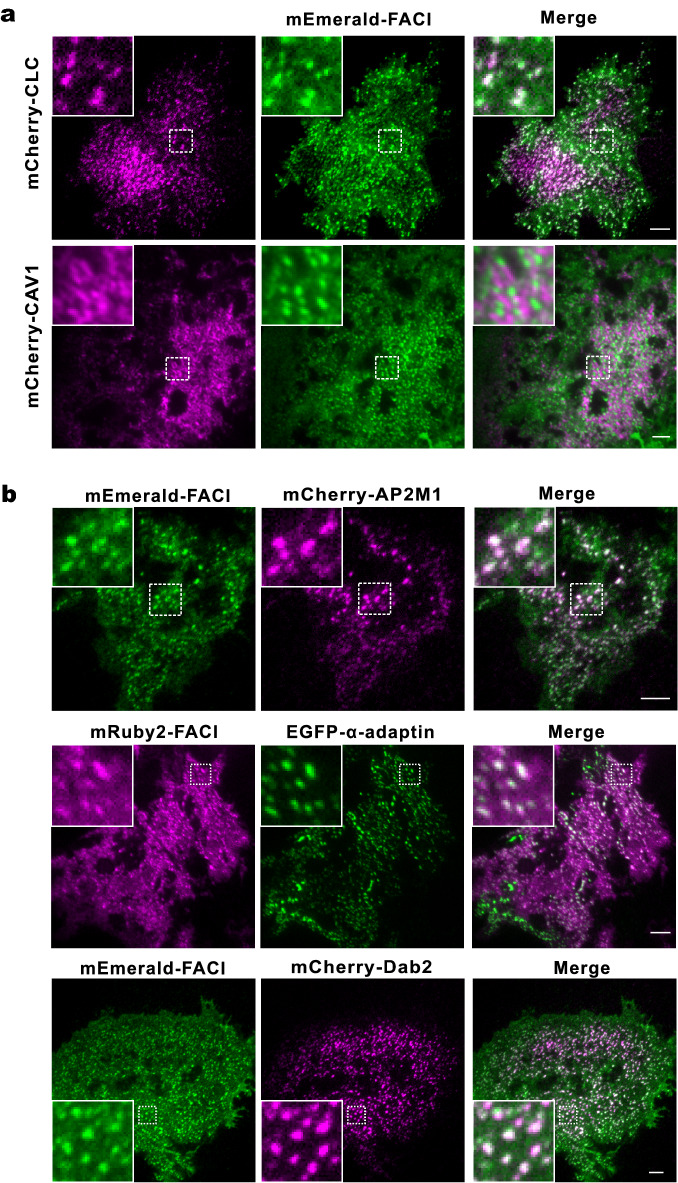
Localization of FACI to CCPs but not caveolae. **A** TIRFM images. AML12 cells expressing mEmerald-FACI were transfected with mCherry-CLC or mCherry-CAV1 plasmid. Scale bar, 5 μm. **B** TIRFM images of AML12 cells expressing mEmerald-FACI and mCherry-AP2M1, mRuby2-FACI and EGFP-α-adaptin, or mEmerald-FACI and mCherry-Dab2. Scale bar, 5 μm

To investigate whether FACI might be involved in CME, colocalization of FACI and clathrin to the PM was examined by total internal reflection fluorescence microscopy (TIRFM). mEmerald-FACI appeared in many punctate structures in the PM, which were perfectly colocalized with clathrin spots in both hepatic AML12 (Fig. 2A) and intestinal Caco-2 cells (Additional file 5: Fig. S2). AML12 imaging results also indicated coincidental appearance or disappearance of clathrin and FACI fluorescence in the PM (Additional file 6: Video S1). These results indicated that FACI localized to CCPs in the PM and might internalize through the CME pathway. Apart from clathrin, colocalization of FACI and caveolin was examined by TIRFM. Since mCherry-caveolin-1 (CAV1) and mEmerald-FACI showed distinct distribution in the PM (Fig. 2A), FACI was unlikely critical to caveolae-mediated endocytosis. Besides clathrin, we also checked for colocalization of FACI with clathrin adaptor proteins in the PM. mEmerald-FACI was co-expressed with Dab2 or AP2 subunits AP2M1 and α-adaptin in AML12. Dual-color TIRFM imaging showed that FACI spots were colocalized with Dab2, AP2M1 and α-adaptin in the PM, lending support to the strong association of FACI with the CME pathway (Fig. 2B).

### Requirement of DxxxLI motif of FACI for colocalization with CCPs

**Fig. 3:**
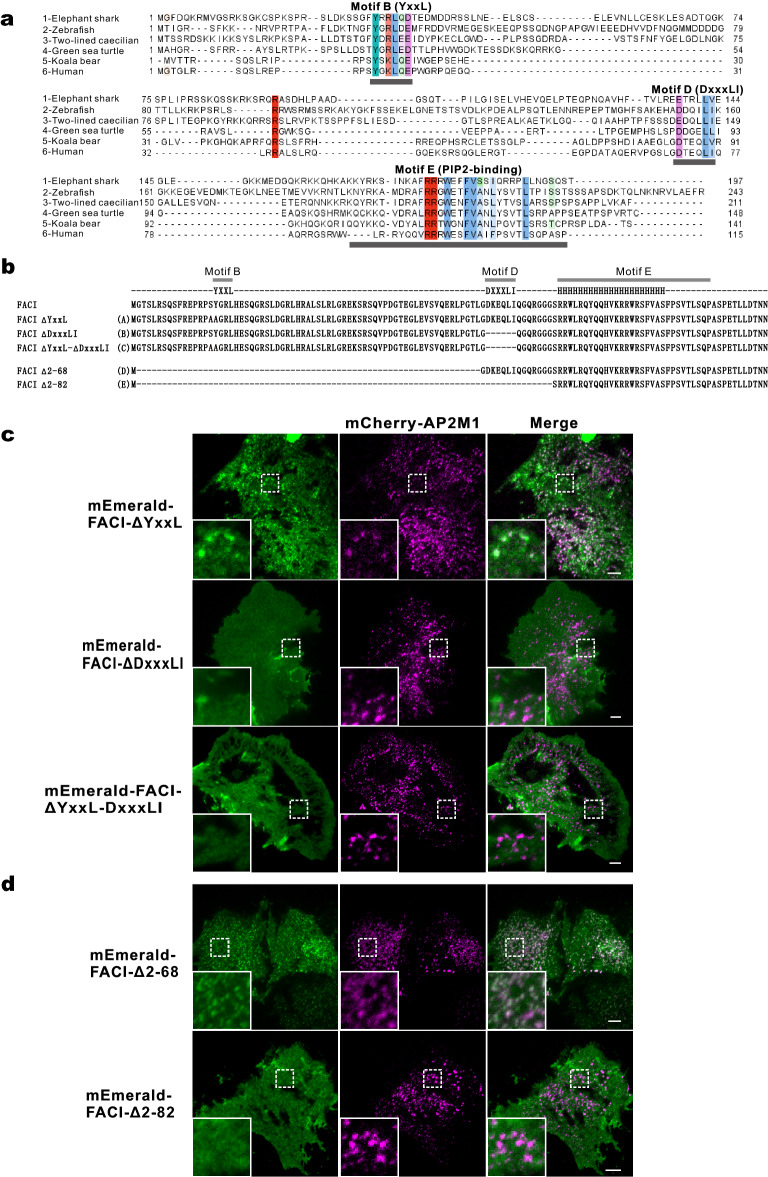
DxxxLI motif of FACI is required for its localization to CCPs. **A** Multiple alignments of FACI and homologs. Conserved Motifs B (YxxL), D (DxxxLI) and E (PIP2-binding motif) were boxed and labeled. FACI homologs were from elephant shark (sb:cb1058, XP_042201215.1), zebrafish (sb:cb1058, NP_001373652.1), two-lined caecilian (LOC115096979, XP_029468198.1), green sea turtle (C11orf86 homolog, XP_027674344.2), koala (C11orf86 homolog, XP_020824020.1) and human (C11orf86, NP_001129957.1). **B** Protein sequences of FACI and its mutants. Motifs B (YxxL), D (DxxxLI) and E (PIP2-binding motif) were highlighted. The amphipathic α-helix of Motif E was represented as a poly-H cluster. **C** TIRFM images of AML12 cells expressing mCherry-AP2M1 and mEmerald-FACI mutants (FACI-ΔYxxL, FACI-ΔDxxxLI, and FACI-ΔYxxL-DxxxLI). Scale bar, 5 µm. **D** TIRFM images of AML12 cells expressing mCherry-AP2M1 and mEmerald-FACI mutants. FACI-Δ2-68 contains Motifs D and E of FACI, while FACI-Δ2-82 just retains Motif E. PIP2: phosphatidylinositol 4,5-bisphosphate. Scale bar, 5 µm

We have previously suggested that the earliest ortholog of FACI might be found in bony fishes [22]. Our further analysis indicated that FACI ortholog should also exist in some cartilaginous fishes (Fig. 3A). Through sequence alignment of all mammalian FACI proteins, we identified five conserved motifs designated A to E. Among the five motifs, Motifs B, D and E are conserved in all FACI orthologs, suggestive of their crucial roles in executing FACI functions (Fig. 3A). We have shown that Motif E is an amphipathic helix-containing motif which binds with plasma phosphoinositides [22]. Motifs B (YxxL) and D (DxxxLI) respectively match the tyrosine-based sorting motif YxxΦ and the acidic dileucine motif [DE]xxxL[LI], both of which are sorting motifs for AP2-mediated endocytosis [3].

We reasoned that the YxxL and DxxxLI motifs of FACI might directly bind with the AP2 complex, which mediates the localization of FACI to CCPs. To test this hypothesis, five mutants of mEmerald-FACI were designed (Fig. 3B). The mEmerald-FACI mutants were co-expressed with mCherry-CLC or AP2M1, and their PM localization was examined by TIRFM. As shown, mEmerald-FACI-ΔDxxxLI and mEmerald-FACI-ΔYxxL-DxxxLI mutants completely lost the punctate localization and instead distributed uniformly in the PM. In keeping with this, the two mutants also lost the colocalization with clathrin and AP2M1 (Fig. 3C and Additional file 7: Video S2). In contrast, the punctate structure in the PM was still evident for mEmerald-FACI-ΔYxxL and its colocalization with clathrin and AP2M1 remained (Fig. 3C and Additional file 7: Video S2). However, compared to vesicles formed by mEmerald-FACI, vesicles formed by mEmerald-FACI-ΔYxxL were slightly smaller and showed decreased fluorescent intensity. The results indicated that the CCP localization of FACI might be accounted for by the DxxxLI motif. Despite not being as crucial as Motif D, the YxxL motif could also affect the recruitment of FACI to CCPs.

We further used another two FACI mutants to explore the role of the DxxxLI motif. The mEmerald-FACI-Δ2-68 mutant contained the DxxxLI Motif D and the membrane-binding Motif E, whereas mEmerald-FACI-Δ2-82 contained the Motif E only (Fig. 3B). As indicated (Fig. 3D and Additional file 8: Video S3), both mEmerald-FACI-Δ2-68 and mEmerald-FACI-Δ2-82 retained their PM localization. However, mEmerald-FACI-Δ2-68 still localized to CCPs in the PM but mEmerald-FACI-Δ2-82 did not. These results further suggested that the DxxxLI Motif D of FACI is required for its localization to CCPs, while Motif E is essential for its PM localization.

We have previously demonstrated that FACI mainly localizes to the PM and ERC in a Motif E-dependent manner [22]. Indeed, confocal images showed that deletion of YxxL and DxxxLI didn’t affect the colocalization of FACI with ERC marker Rab11a. (Additional file 5: Fig. S3A). Although the mEmerald-FACI-Δ2-82 mutant lost its localization to CCPs, its localization to the PM and ERC remained unchanged (Additional file 5: Fig. S3B).

### DxxxIL motif of FACI is a AP2 complex-binding motif

**Fig. 4:**
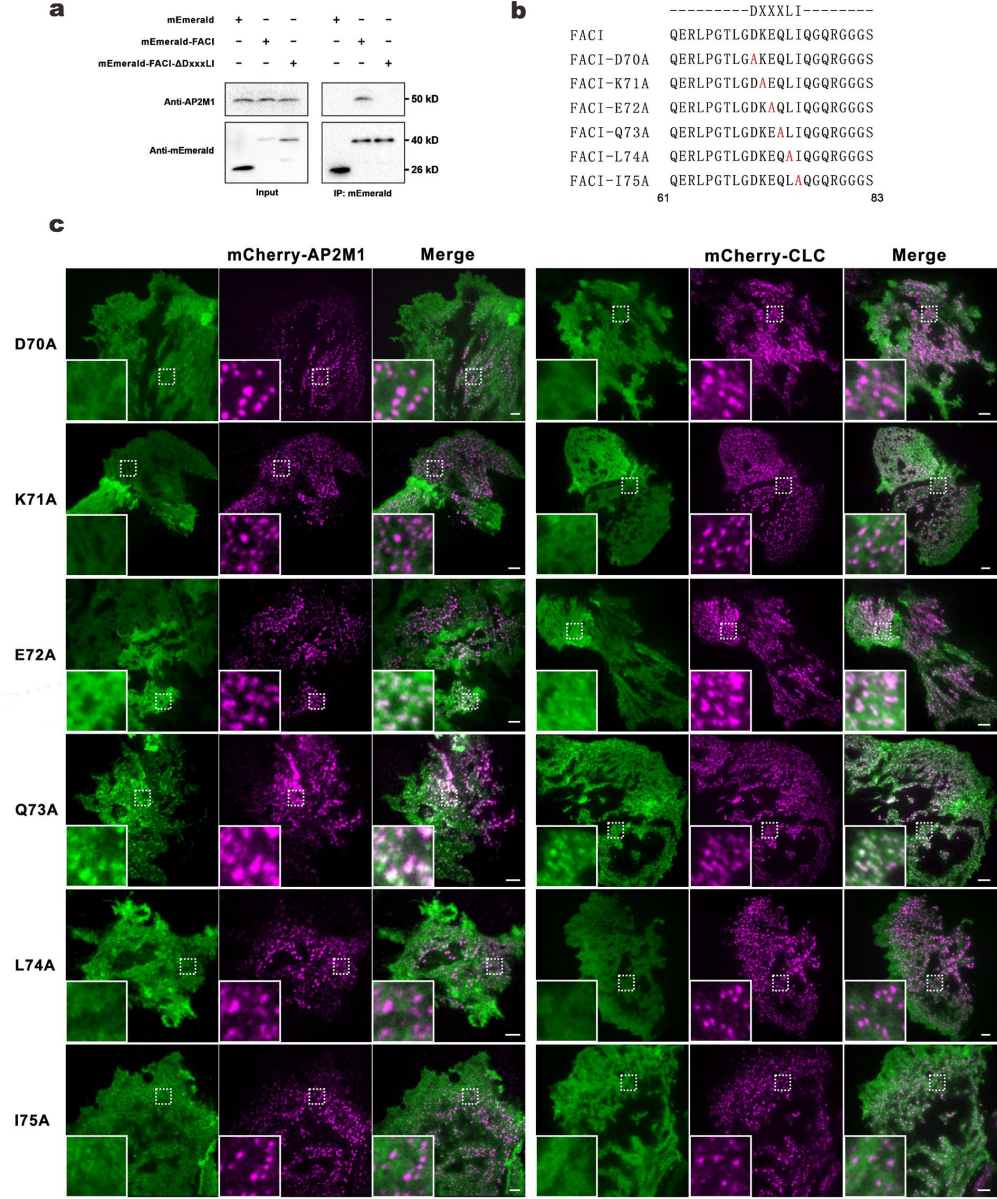
Effects of single-point mutations in DxxxLI motif on FACI’s localization to CCPs.** A** Immunoprecipitation (IP). HepG2 cells were transfected with mEmerald-FACI or mEmerald-FACI-ΔDxxxLI plasmid. Cells were lysed and immune-precipitated with anti-GFP (i.e., anti-mEmerald). Immunoprecipitates were analyzed by SDS-PAGE and probed with anti-GFP and anti-AP2M1 antibodies. **B** DxxxLI motif of FACI proteins was mutated point by point. Amino acid sequences of WT and a series of “DxxxLI”-mutated FACI were shown. **C** TIRFM images. AML12 cells expressing mCherry-AP2M1 (left) or mCherry-CLC (right) were transfected with WT or “DxxxLI”-mutated mEmerald-FACI construct. Scale bar, 5 µm

Our finding indicated the requirement of the Motif D of FACI for its localization to CCPs. Next, we tested whether FACI binds with AP2 through the DXXXLI motif. As shown by immunoprecipitation, FACI strongly interacted with endogenous AP2M1, whereas FACI-ΔDxxxLI lost the ability to bind with AP2M1 (Fig. 4A and Additional file 5: Fig. S4).

In the acidic dileucine motifs [ED]xxxL[LI], the first acidic amino acid (D or E) and the last two amino acids (L or I) are critical for its binding with the α/σ2 heterodimer of AP2 complex [41]. Therefore, we asked which amino acids in the DxxxLI motif (DKEQLI) of FACI might be crucial for its binding with AP2 complex. A series of point mutants with altered DxxxLI motif (DKEQLI) were constructed and tested (Fig. 4B). Notably, mEmerald-FACI-D70A, -K71A, -L74A and -I75A distributed evenly to the PM and completely lost the colocalization with AP2M1 and clathrin. In contrast, mEmerald-FACI-Q73A still formed speckles in the PM and localized to CCPs. mEmerald-FACI-E72A also showed extremely weak colocalization with AP2M1 and clathrin (Fig. 4C). The mutational analysis further confirmed that DxxxLI of FACI protein is an acidic dileucine motif required for FACI binding to the AP2 complex.

### Cholesterol induces PM-to-ERC transport of FACI in a clathrin-dependent manner

Our subcellular localization and interactome analysis of FACI suggested that through dynamic distribution between the PM and endocytic recycling machinery, FACI might play a role in endocytosis and vesicle trafficking. Thus, we further explored the factors affecting intracellular trafficking of FACI. To this end, a stable AML12 cell line inducibly expressing mRuby2-FACI (AML12-mRuby2-FACI) was generated.

**Fig. 5:**
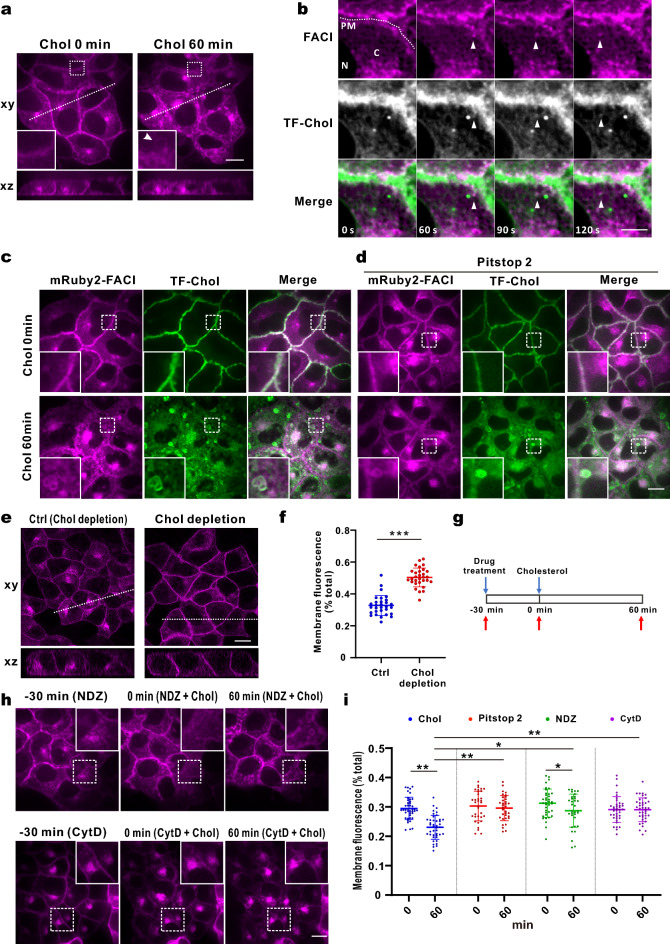
Cholesterol regulates FACI transport from the PM to ERC in a clathrin-dependent manner. **A** Cholesterol loading assay. The AML12-mRuby2-FACI cell line, which stably expresses mRuby2-FACI in a tetracycline-inducible manner, was used in cholesterol (Chol) loading assay. Before the day of the experiment, doxycycline (dox) was added into the medium of AML12-mRuby2-FACI cells to induce mRuby2-FACI expression. For cholesterol loading, cells were treated with 40 µg/mL water-soluble cholesterol for 1 h. Z-stack scans were taken by spinning disc confocal microscopy (SDCM) before and after the cholesterol treatment for the same cells. Representative xy and Z-stack xz images were shown. Scale bar, 10 µm. **B** Time-lapse confocal live-cell imaging. AML12-mRuby2-FACI cells were treated with 40 µg/mL water-soluble cholesterol together with 2 ng/mL TopFluor cholesterol (TF-chol). Images were acquired with a 10 s time interval. The co-translocation of mRuby2-FACI and TopFluor cholesterol from the PM to cytosol was tracked (white arrowhead). Green: TopFluor cholesterol; Magenta: mRuby2-FACI. *N* nucleus. *C* cytoplasm. Scale bar, 5 µm. **C** AML12-mRuby2-FACI cells were treated with 40 µg/mL water-soluble cholesterol together with 2 ng/mL TopFluor cholesterol for 1 h. Images of cells before and after cholesterol treatment were shown. Scale bar, 10 µm.** D** CME blockade assay. AML12-mRuby2-FACI cells were pretreated with Pitstop-2 for 15 min and then incubated with water-soluble cholesterol for 1 h. Images of cells before and after cholesterol treatment were shown. Green: TopFluor cholesterol; Magenta: mRuby2-FACI. Scale bar, 10 µm. **E** Cholesterol depletion assay. Cells were treated with a cholesterol-depleting medium (with 5 mM MβCD) for 2 h. Cells with mock treatment were used as control. Cells were fixed and examined by confocal microscopy. Scale bar, 10 µm. **F** Percentages of intracellular FACI signals against whole cell FACI signals were quantified. n = 30. Scale bar, 10 µm. Ctrl: control. Statistical significance was evaluated by two-tailed unpaired Student’s t test. ***: P < 0.001. **G** Disruption of cytoskeleton impairs FACI trafficking from the PM to ERC. The scheme of drug treatment. Cells were first treated with 2 µM nocodazole (NDZ) or 2 µM cytochalasin D (CytD) for 30 min. Water-soluble cholesterol (40 µg/mL) was then added into the culture media for another 60 min. Images were acquired at the indicated time points (red arrow). **H** Cells were treated as shown in (**G**). Cells were kept on the TOKAI HIT stage-top incubator with 5% CO_2_ at 37 °C and imaged by SDCM at the indicated time points. Scale bar, 10 µm. **I** Quantification of the intracellular mRuby2-FACI fluorescence intensity relative to the intensity of the whole cell. n = 25 ~ 35. Statistical significance was evaluated by one-way ANOVA with Tukey's post hoc tests. *: P < 0.05. ***: P < 0.001. ns: not significant

We found that cholesterol was most influential on intracellular distribution of FACI. In AML12-mRuby2-FACI cells, mRuby2-FACI at steady-state was mainly distributed to the PM and ERC (Fig. 5A, left). Cholesterol loading significantly decreased the intensity of mRuby2-FACI in the PM but increased its intensity on the ERC, indicating that cholesterol could induce FACI transport from the PM to ERC (Fig. 5A, right). Cholesterol loading also relocalized FACI to many large and irregular cytosolic vesicles, which appeared to be early endosome-related membrane structures (Fig. 5A, C). Live cell imaging revealed mRuby2-FACI and TopFluor cholesterol (TF‐Chol, a fluorescent cholesterol tracer) were co-endocytosed from the PM and co-transported in the cytosol (Fig. 5B). Confocal imaging indicated that both mRuby2-FACI and TF-Chol were more intense in the ERC after cholesterol treatment. The FACI-localized “cytosolic vesicles'' were also cholesterol-rich (Fig. 5C). Hence, cholesterol might promote FACI trafficking from the PM to ERC.

We further interrogated whether cholesterol-mediated FACI trafficking might be clathrin-dependent. Pitstop-2, a strong clathrin inhibitor, was used to block CME. Pitstop-2 treatment had minimal effect on the normal distribution of mRuby2-FACI (Additional file 5: Fig. S5A, C). After Pitstop-2 treatment, cholesterol-mediated FACI transport was largely blocked (Fig. 5D, I). The cytosolic vesicles seen in Fig. 5A, C were still observed with decreased numbers. This indicated that cholesterol-mediated FACI transport might be clathrin-dependent.

We next determined the impact of cholesterol depletion on FACI translocation. Cholesterol depletion by methyl-β-cyclodextrin (MβCD) [42] significantly decreased the intensity of mRuby2-FACI in the ERC but increased its intensity in the PM (Fig. 5E). Quantitative analysis further verified an increase in FACI intensity in the PM as a result of cholesterol depletion (Fig. 5F).

Cholesterol plays key roles in both CME and clathrin-independent endocytosis [43–45]. Whereas we cannot exclude the possibility that cholesterol loading and depletion could affect endocytosis, leading to redistribution of FACI, FACI might also directly govern intracellular trafficking of cholesterol between the PM and ERC.

### Disruption of cytoskeleton impairs FACI trafficking from the PM to ERC

Cytoskeleton plays vital roles in endocytosis and vesicle transport [46]. To test if disruption of cytoskeleton would interfere with FACI trafficking, we utilized nocodazole (NDZ) and cytochalasin D (CytD) to inhibit polymerization of microtubules and actin filaments, respectively. Since cholesterol loading rendered FACI internalized and transported to the ERC, we explored how perturbation of cytoskeleton might affect this form of FACI trafficking. As described in Fig. 5G, AML12-mRuby2-FACI cells were pretreated with NDZ or CytD, and then treated with cholesterol to induce mRuby2-FACI translocation from the PM to ERC.

In NDZ-treated cells, the FACI signal vanished from the ERC but slightly increased in the PM (Additional file 5: Fig. S5B, C). This phenomenon was consistent with previous reports [47, 48] and was probably ascribed to disruption of the microtubule structure of ERC by NDZ. In the NDZ- and cholesterol-treated cells, cholesterol still partially induced internalization of mRuby2-FACI (Fig. 5H, I). Internalized mRuby2-FACI failed to move to the ERC but was distributed to many “cytosolic vesicles” (Fig. 5H).

After CytD treatment, the morphology of AML12 cells changed and the cells appeared to be “shrunken”. However, subcellular distribution of mRuby2-FACI was not affected by CytD treatment (Additional file 5: Fig. S5B, C). In the CytD- and cholesterol-treated cells, mRuby2-FACI internalization and transport to ERC was largely blocked (Fig. 5H, I). The ratio of FACI distributed between the PM and the cytosol remained almost constant after CytD administration (Fig. 5I).

Our above findings suggested that the actin cytoskeleton might be indispensable for FACI trafficking from the PM to ERC, whereas microtubule cytoskeleton moderately affected FACI endocytosis but was required for its transport to ERC.

### FACI preferentially facilitates LDL endocytosis

**Fig. 6:**
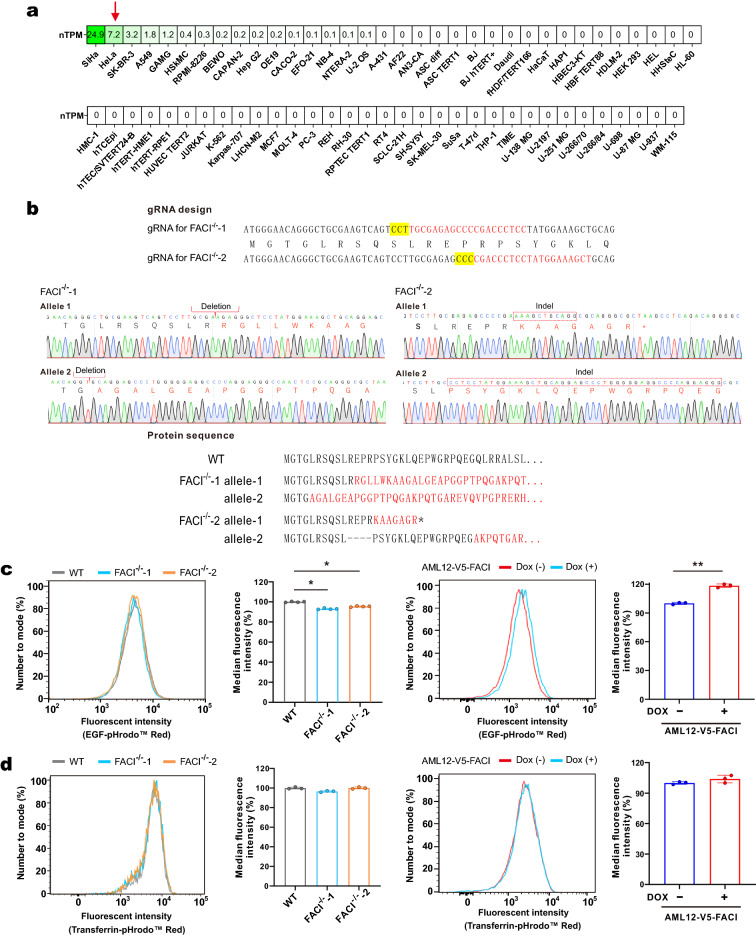
Impact of FACI knockout on EGF and transferrin uptake.** A** FACI mRNA expression intensities in various cell lines. Data were retrieved from NCBI BioProject PRJNA183192. nTPM: normalized transcripts per kilobase million. **B** Generation of FACI^−/−^ HeLa cell lines (FACI^−/−^-1 & FACI^−/−^-2) by CRISPR/Cas9 technique. The region closest to ATG codon of FACI (top) was targeted. gRNAs (red) and PAM sequences (yellow) were highlighted. FACI^−/−^ HeLa cell lines were verified with gene sequencing and representative sequencing chromatographs (middle) were shown. In FACI^−/−^− 1 & FACI^−/−^− 2 cells, the FACI coding regions were disrupted by frameshift and nonsense mutations (bottom). **C** EGF uptake in WT and FACI^−/−^ HeLa cells (left), and in FACI-expressed (Dox) and control (no Dox) AML12 cells (right). The amounts of endocytosed pHrodo-Red-EGF were measured by flow cytometry. The representative flow cytometric histograms and the results of quantitative analysis (n = 3 ~ 4) were indicated. Data are mean fluorescence intensity ± SD. *: P < 0.05. **: P < 0.01 by two-tailed paired Student’s t-test. **D** Transferrin uptake in WT and FACI^−/−^ HeLa cells (left), and in FACI-expressing (Dox) and control (no Dox) AML12 cells (right). The amounts of endocytosed pHrodo-Red-Transferrin were measured by flow cytometry. The representative flow cytometric histograms and the results of quantitative analysis (n = 3 ~ 4) were indicated. Data are mean fluorescence intensity ± SD

A large genome-wide screen indicated that siRNA-mediated depletion of FACI in HeLa cells severely perturbed endocytosis of both epidermal growth factor (EGF) and transferrin (Tfn) [49]. The screening results prompted us to explore whether FACI might play roles in endocytosis. FACI is abundantly expressed in HeLa cells [50], we therefore chose them as the cellular model for loss-of-function study of the role of FACI in endocytosis (Fig. 6A). Two FACI^−/−^ HeLa cell lines FACI^−/−^-1 and FACI^−/−^-2 were established by CRISPR-Cas9 technique. Guide RNAs (gRNAs) targeting the sequence closest to the ATG start codon of FACI were designed for gene editing (Fig. 6B, top). FACI^−/−^ HeLa cell lines were verified by DNA sequencing. The sequencing chromatograph and the disrupted FACI protein sequence were presented (Fig. 6B, middle and bottom). EGF and transferrin endocytosis was compared between FACI^−/−^ and WT HeLa cell lines. FACI^−/−^ HeLa cells showed mildly decreased EGF endocytosis and similar transferrin uptake compared with WT HeLa cells (Fig. 6C, D, left). In keeping with this, FACI overexpression increased EGF endocytosis but showed modest effect on transferrin uptake in AML12 cells (Fig. 6C, D, right).

**Fig. 7:**
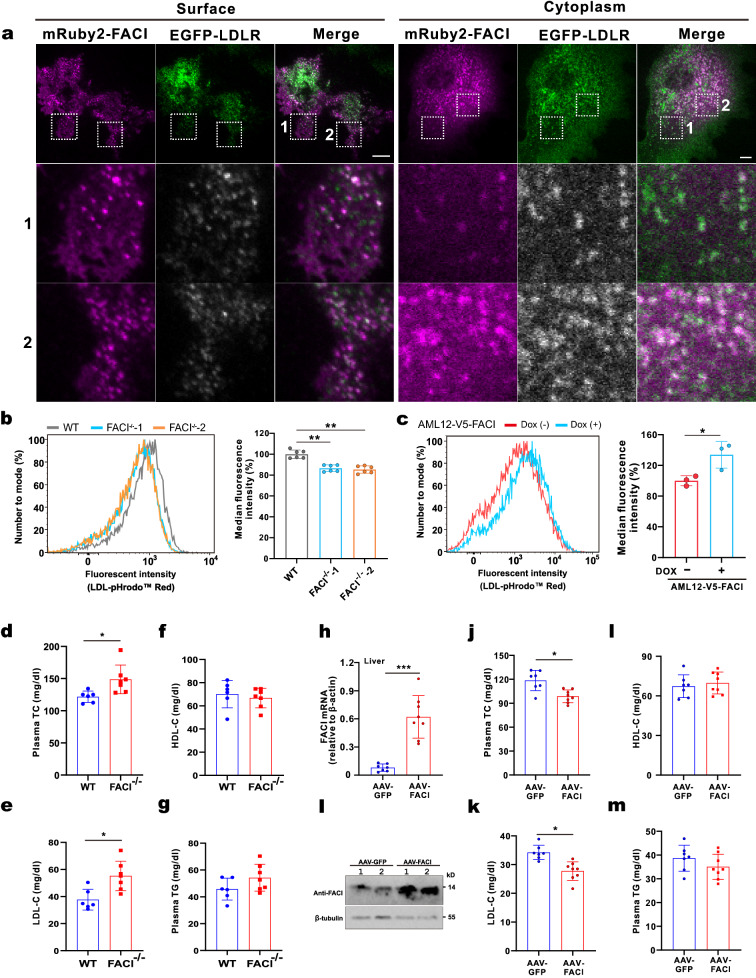
FACI promotes cellular LDL uptake. **A** Images of TIRFM (left) and spinning disc microscopy (right) showing the colocalization of mRuby2-FACI and EGFP-LDLR to PM and cytosol, respectively. Scale bar, 10 μm. **B** Fluorescent LDL uptake assay in WT and FACI^−/−^ HeLa cell lines. The amounts of endocytosed pHrodo-Red-LDL were measured by flow cytometry. A representative flow cytometric histogram shows pHrodo-Red-LDL uptake in WT and FACI^−/−^ HeLa cell lines (left). Quantitative analysis of pHrodo-Red-LDL uptake was performed in replicated experiments (right). Data are mean fluorescence intensity ± SD. **: P < 0.01 by two-tailed paired Student’s t-test. **C** Fluorescent LDL uptake was compared between FACI-expressed (Dox group) and control (no Dox group) AML12 cells. The amounts of endocytosed pHrodo-Red-LDL were measured by flow cytometry. The representative flow cytometric histogram (left) and quantitative analysis (right) were indicated. Data are mean fluorescence intensity ± SD. *: P < 0.05 by two-tailed paired Student’s t-test. **D-G** C57BL/6 J mice (8 weeks old, n = 6) and FACI-/- mice (8 weeks old, n = 7) were fed with a high-cholesterol diet for 4 weeks. Plasma total cholesterol (TC; **D**), LDL-cholesterol (LDL-C; **E**), HDL-cholesterol (HDL-C; **F**) and triglyceride (TG; **G**) were compared. *: P < 0.05 by two-tailed paired Student’s t-test. **H-I** C57BL/6 J mice (6 weeks old) were injected with AAV-FACI (n = 8) or AAV-GFP (n = 7). AAV-FACI is an adeno-associated recombinant virus (AAV) expressing FACI driven by a liver-specific promoter and enhancers. After two-week transduction, mice were fed with a high-cholesterol diet for another 4 weeks. Mice were sacrificed and liver total mRNA was extracted. FACI expression in livers was analyzed by RT-qPCR (**H**) and immunoblotting (**I**). Plasma total cholesterol (TC; **J**), LDL-cholesterol (LDL-C; **K**), HDL-cholesterol (HDL-C; **L**) and triglyceride (TG; **M**) were compared between AAV-FACI and AAV-GFP mice. *: P < 0.05. ***: P < 0.001 by two-tailed paired Student’s t-test

FACI deficiency aggravated hypercholesterolemia implicating that FACI might affect cellular LDL uptake. To shed light on this, colocalization analysis by TIRFM and confocal imaging was performed. In hepatic AML12 cells, mRuby2-FACI and EGFP-LDLR substantially colocalized with each other in both the PM and cytoplasm (Fig. 7A and Additional file 9: Video S4). Compared to WT HeLa, LDL uptake declined in FACI^−/−^ HeLa cell lines (Fig. 7B). To the contrary, a remarkable spike of LDL uptake was observed in stable AML12-V5-FACI cells overexpressing FACI (Fig. 7C). These results indicated that FACI preferentially facilitates LDL endocytosis.

The impact of FACI on LDL endocytosis was further tested in vivo. FACI^−/−^ and WT mice were fed with a high-cholesterol diet for one month and then used for metabolic evaluation of lipids. FACI^−/−^ mice showed higher plasma total cholesterol (TC) and LDL-C levels than WT mice (Fig. 7D, E). High-density lipoprotein-cholesterol (HDL-C) levels were similar between FACI^−/−^ and WT mice (Fig. 7F). Plasma triglycerides (TG) and liver lipids were slightly increased in FACI^−/−^ mice relative to WT mice, which should be caused by enhanced intestinal lipid absorption in FACI^−/−^ mice [22] (Fig. 7G and Additional file 5: Fig. S6A).

We also investigated whether FACI promotes LDL endocytosis in FACI-overexpression mice. Liver is the main organ involved in clearing circulating LDL cholesterol. A liver-directed adeno-associated virus (AAV) expression system was used to persistently overexpress hepatic FACI in mice. Liver-specific expression of FACI by AAV also avoided any side effects caused by intestinal FACI. The 6-week-old mice (male) were randomly divided into two groups: AAV-GFP group infected with AAV expressing EGFP and AAV-FACI group infected with AAV that expresses FACI. After two weeks of infection, mice were fed with a high-cholesterol diet for another month and then used for metabolic evaluation of lipids. Real-time qPCR results confirmed successful overexpression of exogenous FACI via AAV transduction (Additional file 5: Fig. S6B). In keeping with this, the transcript and protein levels of total FACI were substantially increased in livers of AAV-FACI-infected mice (Fig. 7H, I). Lipid profile test indicated a drop in plasma total cholesterol and LDL-C levels in AAV-FACI mice relative to control mice (Fig. 7J, K). Plasma HDL-cholesterol, total triglyceride, hepatic cholesterol and hepatic triglyceride concentrations were however unaffected by the overexpression of FACI (Fig. 7L, M and Additional file 5: Fig. S6C).

## Discussion

We have previously characterized biochemical properties of FACI including tissue distribution, subcellular localization, gene evolution and transcriptional regulation. We also found that *Faci*^−/−^ mice are prone to develop diet-induced hypercholesterolemia [22]. In this study, we further analyzed the interactome of FACI (Fig. 1) and found that FACI might be involved in CME (Fig. 2). The DxxxLI motif (Motif D) of FACI protein directly interacts with the AP2 complex to exert its impact on CME (Figs. 3, 4). Cholesterol induced FACI transport from the PM to ERC in a clathrin-dependent manner (Fig. 5). FACI preferentially affected LDL uptake but had modest effect on EGF endocytosis (Fig. 6 and Fig. 7). Importantly, diet-induced hypercholesterolemia in mice was alleviated by hepatic overexpression of FACI (Fig. 7).

**Fig. 8:**
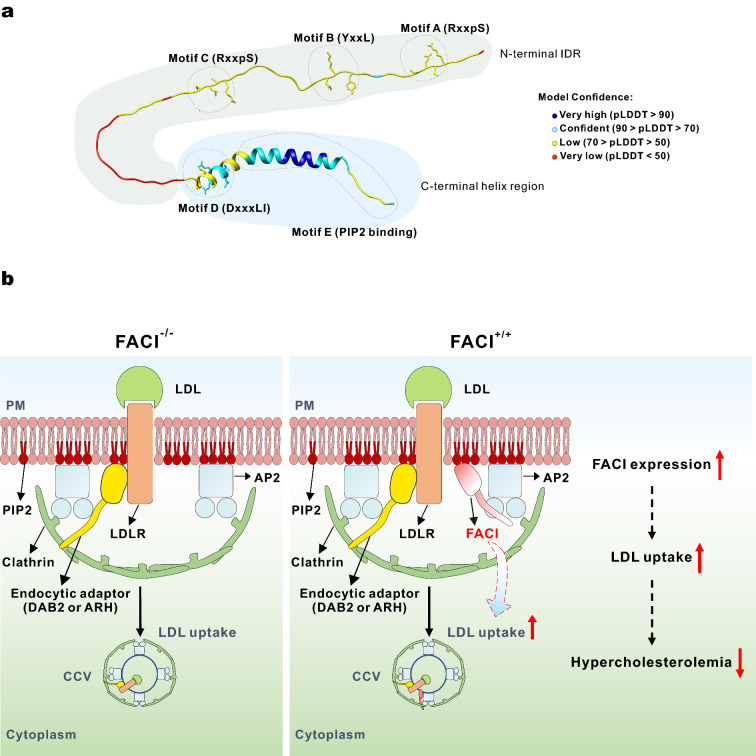
Schematic diagram showing the predicted 3D structure of FACI and its role on clathrin-mediated LDLR endocytosis.** A** Predicted 3D structure of FACI by AlphaFold2 [59]. FACI protein is composed of two parts: N-terminal intrinsic disorder region (IDR) and C-terminal helix region. C-terminal helix region consists of Motifs D (DxxxLI) and E (PIP2-binding motif). N-terminal IDR contains Motifs A (RxxpS), B (YxxL) and C (RxxpS). Motifs A and C are two phosphorylated motifs with unknown function, which are conserved among mammalian FACI homologs. pLDDT: per-residue confidence score. Regions below 50 pLDDT may be unstructured in isolation. **B** The role of FACI in clathrin-mediated LDLR endocytosis. FACI is an integral monotopic protein or a peripheral membrane protein. Its C-terminal amphipathic α-helix-containing motif (Motif E) mediates the binding with membrane phosphatidylinositol, while the N-terminal IDR is “string-like” in the cytoplasm. Under some conditions, FACI is recruited by membrane PIP2. PIP2-bound FACI further recruits the AP2 complex by its DxxxLI motif (Motif D), which drives or promotes assembly of CCVs. FACI increases the efficiency of LDLR endocytosis, which attenuates diet-induced hypercholesterolemia in mice

Based on our findings on FACI, we proposed the following model (Fig. 8A). FACI is a small protein with about 115 amino acids without any known functional domain. FACI protein consists of two parts: N-terminal intrinsic disorder region (IDR) and C-terminal helix region. The C-terminal helix region contains the DxxxLI motif (Motif D) that binds directly with the AP2 complex, and the amphipathic helix-containing motif (Motif E), which binds with membrane phosphatidylinositol. The N-terminal IDR harbors three evolutionarily conserved short linear motifs (Motifs A to C). Motif B (YxxL) appears to be another recognition motif of AP2, although it is not as crucial as Motif D. Motifs A and C are two phosphorylated motifs [51]. They have a similar sequence pattern RXXpS, which matches the recognition sites of some AGC kinases such as PKA, PKB and PKC [52]. The exact function of Motifs A and C remains elusive.

FACI is an integral monotopic protein or a peripheral membrane protein, which is attached to the inner leaflet of PM and the outer leaflet of the endosomal membrane (Fig. 8B). Motif E is responsible for membrane binding, and the N-terminal IDR is string-like in the cytoplasm. Under certain conditions, FACI is recruited by membrane PIP2. PIP2-bound FACI interacts with the AP2 complex through its DxxxLI Motif D, which further leads to the recruitment of clathrin and other endocytic proteins to form CCPs. FACI, through an unknown mechanism, promotes the sorting of LDLR into CCPs. FACI in the CCPs is transported into EE, and finally returned to the PM through the Rab11-dependent recycling pathway [22].

FACI protein shares structural similarities with many endocytic adaptors, such as Epsins 1 to 3 and phosphotyrosine-binding (PTB)-domain containing adaptors ARH, Dab2 and Numb. These proteins adapt transmembrane cargos to the endocytic machinery. They share two structural properties. First, they bind all or some key components of the endocytic machinery including PIP2, cargos, clathrin, accessory proteins and adaptor proteins [5]. Second, they usually have a folded domain located at one of their termini, while their remaining region is IDR with embedded functional motifs [53]. For example, the Epsin adaptor starts off with an N-terminal epsin N-terminal homology (ENTH) domain followed by a long IDR. The ENTH domain serves as a PIP2-binding module, while the long IDR contains cargo-binding motifs, AP2-binding motifs, and clathrin-binding motifs. The PTB-domain containing adaptors have an N-terminal PTB domain which mediates PIP2 binding and cargo recognition, and a long unstructured C-terminal tail which harbors AP2-binding motifs and clathrin-binding motifs [6]. FACI protein consists of a long N-terminal IDR embedded with Motifs A to C, and C-terminal helix region for its PIP2- and AP2-binding. FACI deficiency in cells impaired LDL uptake and modestly affected EGF endocytosis. Therefore, we speculate that FACI is probably a novel enterocyte- and hepatocyte-enriched endocytic adaptor.

Our work has derived a new understanding of the function of the C-terminal helical region of FACI. However, the exact function of its IDR remains to be clarified. One probable function of this IDR could be recruitment of endocytic cargos, endocytic accessory proteins, or other proteins through Motifs A to C. Because of the flexibility of IDRs, Motifs A to C can be easily accessed and recognized by the binding partners. In particular, Motifs A and C are two phosphorylated motifs. Phosphorylation at these sites might serve key regulatory roles. In addition, recent studies indicated that IDRs of endocytic adaptor proteins, such as Epsin 1 and AP180, might drive the curvature of CCPs [54]. Whether FACI IDR might regulate membrane curvature merits further analysis.

LDL uptake is crucial for cholesterol homeostasis. Our study uncovered a novel function of FACI in LDLR endocytosis. Diet-induced hypercholesterolemia was aggravated in *Faci*^−/−^ mice but alleviated in hepatic FACI-overexpressed mice. Exactly how FACI regulates LDL uptake remains to be elucidated. FACI might facilitate the recruitment of LDLR into CCVs by reinforcing the interaction between AP2 and ARH or enhancing Dab2 function through unknown mechanisms. In addition, in light of the strong colocalization between FACI and LDLR, FACI could also enhance endocytic recycling of LDLR.

We have characterized the role of FACI in LDLR endocytosis in this study. However, several areas concerning the exact endocytic function of FACI remain to be further explored. First, FACI is highly expressed in enterocytes. Intestinal LDL uptake has been shown to mediate TICE [55]. FACI might be engaged in the TICE process. Second, intestinal cholesterol absorption is mediated through clathrin-dependent NPC1L1 endocytosis [56]. The influence of FACI on endocytosis and recycling of NPC1L1 is worthy of further investigation. Finally, as a potential endocytic adaptor, FACI plausibly affects endocytosis and recycling of other membrane proteins in addition to LDLR. In this regard, the FACI interactome study has provided a good starting point for identification of other FACI-specific cargos.

FACI was found to facilitate LDLR endocytosis preferentially. The impact of FACI knockout on endocytosis of EGFR and transferrin was more modest. This was not surprising as similar preference has also been demonstrated for other endocytic adaptors such as Dab2 [12] and ARH [57]. Plausibly, FACI might function either in a similar fashion or through some of these adaptors.

Blocking LDL uptake has been a very successful strategy for rational design and development of cholesterol-lowering agents for clinical use. PCSK9 inhibitors have proved beneficial in large clinical trials [58]. In this regard, elucidation of the role of FACI in LDLR endocytosis might reveal novel targets and strategies for prevention and treatment of dyslipidemia.

## Supplementary Information

**Additional file 1: Table S1.** Lists of reagents, plasmids, antibodies, and oligonucleotides.

**Additional file 2: Table S2.** BioID-mass spectrometric data. Lists of potential FACI-interacting proteins in AML12 identified with the BioID approach.

**Additional file 3: Table S3.** Affinity purification-mass spectrometric data. Lists of potential FACI-interacting proteins in AML12 identified with affinity purification approach.

**Additional file 4: Table S4.** Lists of 32 FACI interactors based on stringent prediction criteria and their gene ontology analysis.

**Additional file 5: Figure S1. **Generation of stable AML12 cells. AML12 cell lines stably expressing V5-FACI (AML12-V5-FACI) were generated and verified by immunofluorescence. A mock AML12 stable cell line (AML12-CTR) was generated and used as a control. Scale bar, 10 µm.** Fig. S2.** FACI localizes to CCPs in Caco-2 cells. TIRFM images. Caco-2 cells expressing mEmerald-FACI were transfected with mCherry-CLC plasmids. Scale bar, 5 μm.** Fig. S3.** Deletion of DxxxLI and YxxL motifs of FACI does not affect its localization to PM and ERC. (A) Confocal images of AML12 cells expressing mCherry-Rab11a and mEmerald-FACI mutants (FACI-ΔYxxL, FACI-ΔDxxxLI, and FACI-ΔYxxL-DxxxLI). Scale bar, 20 µm. (B) Confocal images of AML12 cells expressing mCherry-Rab11a and mEmerald-FACI mutants (FACI-Δ2-68 and FACI-Δ2-82). Scale bar, 10 µm. **Fig. S4.** AML12 cells expressing mCherry-AP2M1 were transfected with mEmerald-FACI or mEmerald-FACI-ΔDxxxLI plasmid. Cells were lysed and immunoprecipitated with anti-mCherry. Immunoprecipitates were analyzed by SDS-PAGE and probed with the indicated antibodies. **Fig. S5.** (A,B) AML12-mRuby2-FACI stable cells were incubated with Pitstop-2 (A), NDZ (B, upper panel) or CytD (B, lower panel) for the indicated time periods. Cells were kept on the TOKAI HIT stage-top incubator with 5% CO_2_ at 37°C of the microscope and imaged by SDCM at the indicated time points. Scale bar, 10 µm. (C) Quantification of the intracellular mRuby2-FACI fluorescence intensity relative to the intensity of the whole cell before (Ctrl 0 min) and after drug treatment (NDZ 90 min, CytD 90 min or Pitstop-2 75 min). n = 25-35. Statistical significance was evaluated by one-way ANOVA with Tukey's post hoc tests.** Fig. S6.** **A **C57BL/6J mice (8 weeks old, n=6) and FACI-/- mice (8 weeks old, n=7) were fed with a high-cholesterol diet for 4 weeks. Body weights, liver weights, liver cholesterol and liver triglyceride contents of mice were measured. **B **C57BL/6J mice (6 weeks old) were injected with AAV-FACI (n = 8) or AAV-GFP (n = 7). After two-week transduction, mice were fed with a high-cholesterol diet for another 4 weeks. AAV-mediated exogenous FACI expression in livers was analyzed by RT-qPCR. **C **Body weights, liver weights, liver cholesterol and liver triglyceride contents of mice in **B** were measured.

**Additional file 6: Video S1.** Bottom surface of an AML12 cell expressing mEmerald-FACI and mCherry-CLC was imaged by TIRFM.

**Additional file 7: Video S2.** Bottom surfaces of AML12 cells expressing mEmerald-FACI-ΔDxxxLI and mCherry-AP2M1 as well as AML12 cells expressing mEmerald-FACI-ΔYxxL and mCherry-AP2M1 were imaged by TIRFM.

**Additional file 8: Video S3.** Bottom surfaces of an AML12 cells expressing mEmerald-FACI-Δ2-68 and mCherry-AP2M1 as well as AML12 cells expressing FACI-Δ2-82 and mCherry-AP2M1 were imaged by TIRFM. FACI-Δ2-68 contains Motifs D and E of FACI, while FACI-Δ2-82 contains Motif E.

**Additional file 9: Video S4.** Live imaging showing the colocalization of mRuby2-FACI and EGFP-LDLR in cytosol. AML12 cells transfected with mRuby2-FACI and EGFP-LDLR were imaged by spinning disc microscopy.

## Data Availability

The datasets generated during the current study are available from the corresponding authors on reasonable request.
