# Supplementary figures and images for: FACI is a novel clathrin adaptor protein 2-binding protein that facilitates low-density lipoprotein endocytosis
Source: Cell Biosci. 2023 Apr 18;13:74. doi: 10.1186/s13578-023-01023-5 (PMC10114425; doi:10.1186/s13578-023-01023-5)

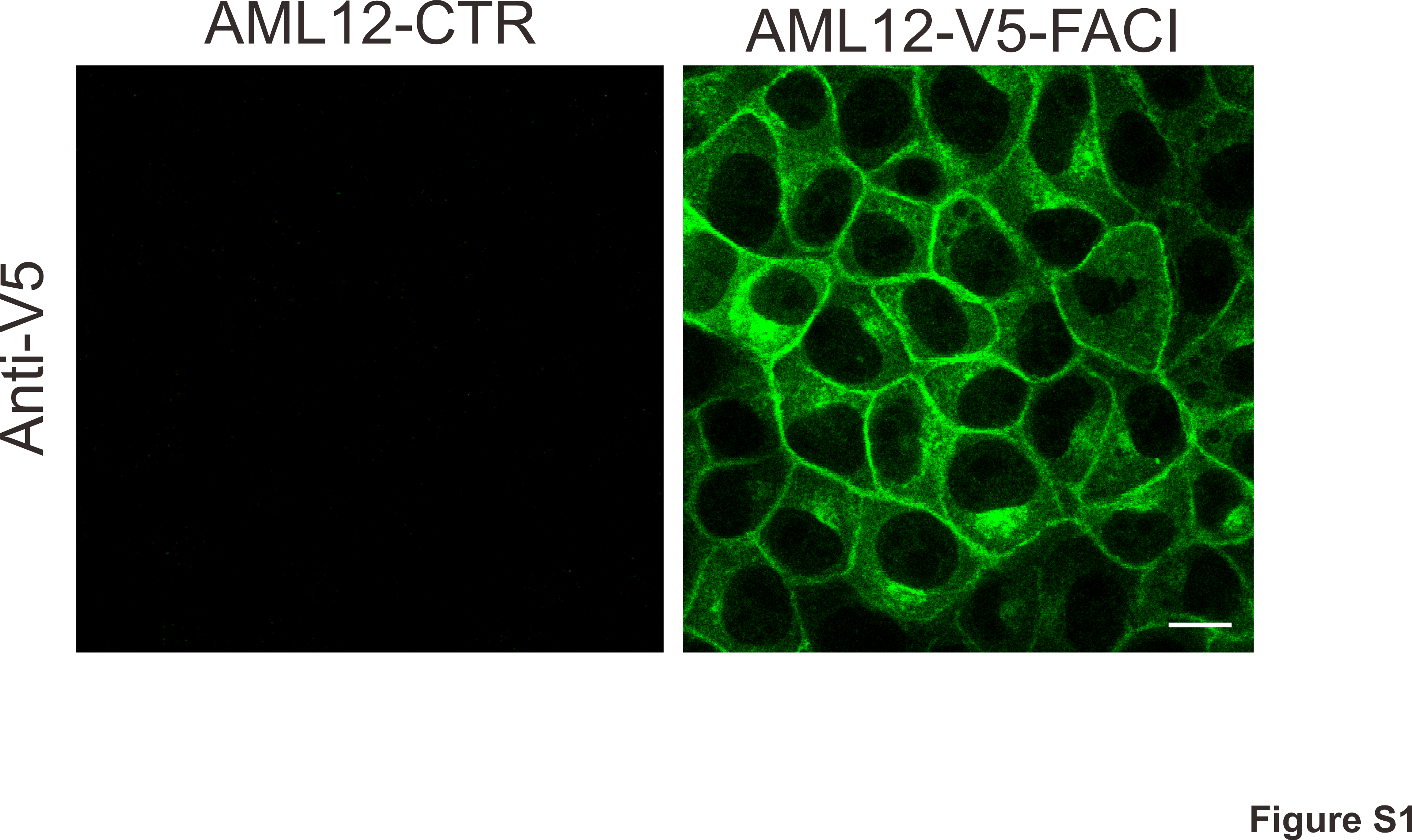

Supplement: Supplementary file 5 — Additional file 5: Figure S1. Generation of stable AML12 cells. AML12 cell lines stably expressing V5-FACI (AML12-V5-FACI) were generated and verified by immunofluorescence. A mock AML12 stable cell line (AML12-CTR) was generated and used as a control. Scale bar, 10 µm. Fig. S2. FACI localizes to CCPs in Caco-2 cells. TIRFM images. Caco-2 cells expressing mEmerald-FACI were transfected with mCherry-CLC plasmids. Scale bar, 5 μm. Fig. S3. Deletion of DxxxLI and YxxL motifs of FACI does not affect its localization to PM and ERC. (A) Confocal images of AML12 cells expressing mCherry-Rab11a and mEmerald-FACI mutants (FACI-ΔYxxL, FACI-ΔDxxxLI, and FACI-ΔYxxL-DxxxLI). Scale bar, 20 µm. (B) Confocal images of AML12 cells expressing mCherry-Rab11a and mEmerald-FACI mutants (FACI-Δ2-68 and FACI-Δ2-82). Scale bar, 10 µm. Fig. S4. AML12 cells expressing mCherry-AP2M1 were transfected with mEmerald-FACI or mEmerald-FACI-ΔDxxxLI plasmid. Cells were lysed and immunoprecipitated with anti-mCherry. Immunoprecipitates were analyzed by SDS-PAGE and probed with the indicated antibodies. Fig. S5. (A,B) AML12-mRuby2-FACI stable cells were incubated with Pitstop-2 (A), NDZ (B, upper panel) or CytD (B, lower panel) for the indicated time periods. Cells were kept on the TOKAI HIT stage-top incubator with 5% CO2 at 37°C of the microscope and imaged by SDCM at the indicated time points. Scale bar, 10 µm. (C) Quantification of the intracellular mRuby2-FACI fluorescence intensity relative to the intensity of the whole cell before (Ctrl 0 min) and after drug treatment (NDZ 90 min, CytD 90 min or Pitstop-2 75 min). n = 25-35. Statistical significance was evaluated by one-way ANOVA with Tukey's post hoc tests. Fig. S6. A C57BL/6J mice (8 weeks old, n=6) and FACI-/- mice (8 weeks old, n=7) were fed with a high-cholesterol diet for 4 weeks. Body weights, liver weights, liver cholesterol and liver triglyceride contents of mice were measured. B C57BL/6J mice (6 weeks old) were injected with AAV-FACI [file 13578_2023_1023_MOESM5_ESM.zip › supplementary images/FigS1-12Mar.tif]

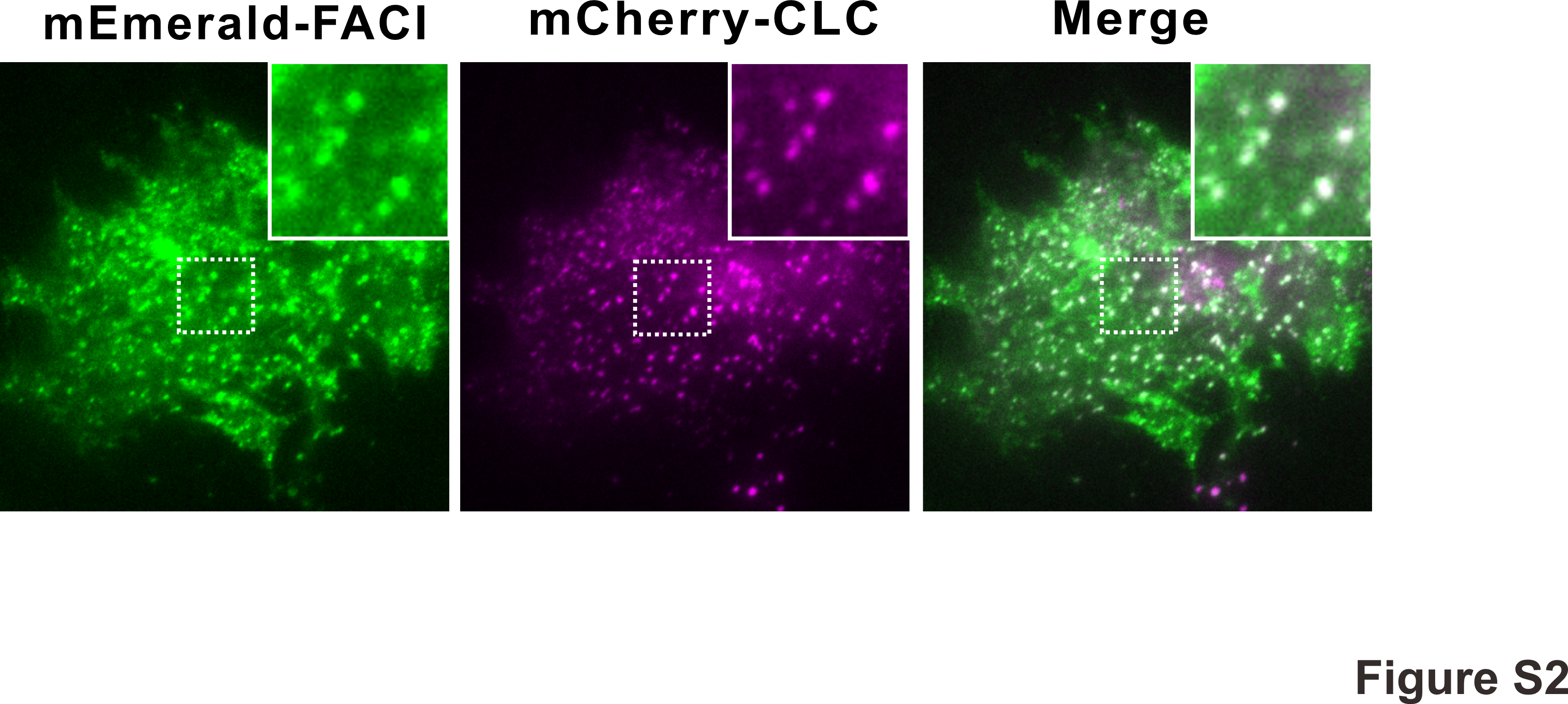

Supplement: Supplementary file 5 — Additional file 5: Figure S1. Generation of stable AML12 cells. AML12 cell lines stably expressing V5-FACI (AML12-V5-FACI) were generated and verified by immunofluorescence. A mock AML12 stable cell line (AML12-CTR) was generated and used as a control. Scale bar, 10 µm. Fig. S2. FACI localizes to CCPs in Caco-2 cells. TIRFM images. Caco-2 cells expressing mEmerald-FACI were transfected with mCherry-CLC plasmids. Scale bar, 5 μm. Fig. S3. Deletion of DxxxLI and YxxL motifs of FACI does not affect its localization to PM and ERC. (A) Confocal images of AML12 cells expressing mCherry-Rab11a and mEmerald-FACI mutants (FACI-ΔYxxL, FACI-ΔDxxxLI, and FACI-ΔYxxL-DxxxLI). Scale bar, 20 µm. (B) Confocal images of AML12 cells expressing mCherry-Rab11a and mEmerald-FACI mutants (FACI-Δ2-68 and FACI-Δ2-82). Scale bar, 10 µm. Fig. S4. AML12 cells expressing mCherry-AP2M1 were transfected with mEmerald-FACI or mEmerald-FACI-ΔDxxxLI plasmid. Cells were lysed and immunoprecipitated with anti-mCherry. Immunoprecipitates were analyzed by SDS-PAGE and probed with the indicated antibodies. Fig. S5. (A,B) AML12-mRuby2-FACI stable cells were incubated with Pitstop-2 (A), NDZ (B, upper panel) or CytD (B, lower panel) for the indicated time periods. Cells were kept on the TOKAI HIT stage-top incubator with 5% CO2 at 37°C of the microscope and imaged by SDCM at the indicated time points. Scale bar, 10 µm. (C) Quantification of the intracellular mRuby2-FACI fluorescence intensity relative to the intensity of the whole cell before (Ctrl 0 min) and after drug treatment (NDZ 90 min, CytD 90 min or Pitstop-2 75 min). n = 25-35. Statistical significance was evaluated by one-way ANOVA with Tukey's post hoc tests. Fig. S6. A C57BL/6J mice (8 weeks old, n=6) and FACI-/- mice (8 weeks old, n=7) were fed with a high-cholesterol diet for 4 weeks. Body weights, liver weights, liver cholesterol and liver triglyceride contents of mice were measured. B C57BL/6J mice (6 weeks old) were injected with AAV-FACI [file 13578_2023_1023_MOESM5_ESM.zip › supplementary images/FigS2-12Mar.tif]

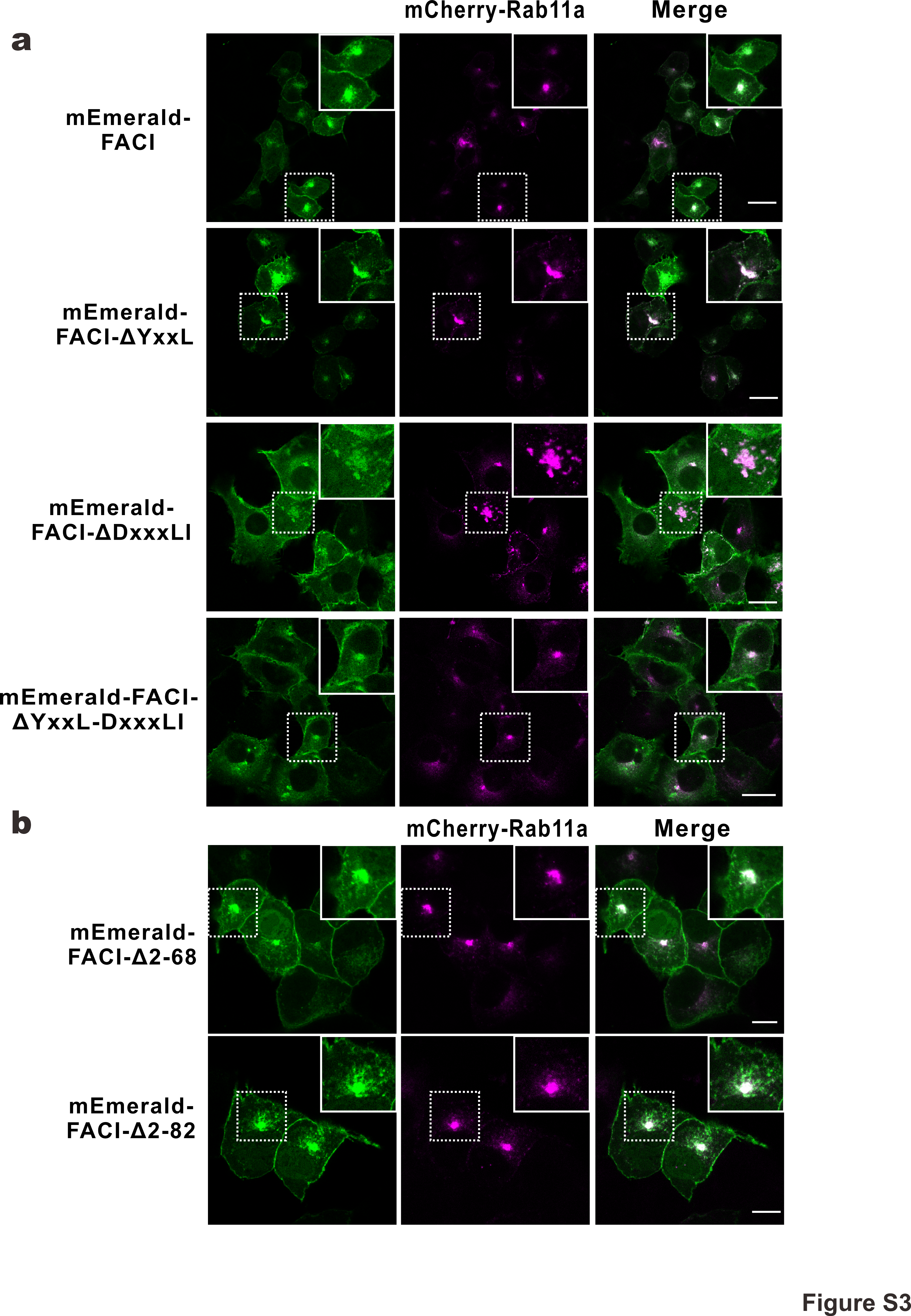

Supplement: Supplementary file 5 — Additional file 5: Figure S1. Generation of stable AML12 cells. AML12 cell lines stably expressing V5-FACI (AML12-V5-FACI) were generated and verified by immunofluorescence. A mock AML12 stable cell line (AML12-CTR) was generated and used as a control. Scale bar, 10 µm. Fig. S2. FACI localizes to CCPs in Caco-2 cells. TIRFM images. Caco-2 cells expressing mEmerald-FACI were transfected with mCherry-CLC plasmids. Scale bar, 5 μm. Fig. S3. Deletion of DxxxLI and YxxL motifs of FACI does not affect its localization to PM and ERC. (A) Confocal images of AML12 cells expressing mCherry-Rab11a and mEmerald-FACI mutants (FACI-ΔYxxL, FACI-ΔDxxxLI, and FACI-ΔYxxL-DxxxLI). Scale bar, 20 µm. (B) Confocal images of AML12 cells expressing mCherry-Rab11a and mEmerald-FACI mutants (FACI-Δ2-68 and FACI-Δ2-82). Scale bar, 10 µm. Fig. S4. AML12 cells expressing mCherry-AP2M1 were transfected with mEmerald-FACI or mEmerald-FACI-ΔDxxxLI plasmid. Cells were lysed and immunoprecipitated with anti-mCherry. Immunoprecipitates were analyzed by SDS-PAGE and probed with the indicated antibodies. Fig. S5. (A,B) AML12-mRuby2-FACI stable cells were incubated with Pitstop-2 (A), NDZ (B, upper panel) or CytD (B, lower panel) for the indicated time periods. Cells were kept on the TOKAI HIT stage-top incubator with 5% CO2 at 37°C of the microscope and imaged by SDCM at the indicated time points. Scale bar, 10 µm. (C) Quantification of the intracellular mRuby2-FACI fluorescence intensity relative to the intensity of the whole cell before (Ctrl 0 min) and after drug treatment (NDZ 90 min, CytD 90 min or Pitstop-2 75 min). n = 25-35. Statistical significance was evaluated by one-way ANOVA with Tukey's post hoc tests. Fig. S6. A C57BL/6J mice (8 weeks old, n=6) and FACI-/- mice (8 weeks old, n=7) were fed with a high-cholesterol diet for 4 weeks. Body weights, liver weights, liver cholesterol and liver triglyceride contents of mice were measured. B C57BL/6J mice (6 weeks old) were injected with AAV-FACI [file 13578_2023_1023_MOESM5_ESM.zip › supplementary images/FigS3-12Mar.tif]

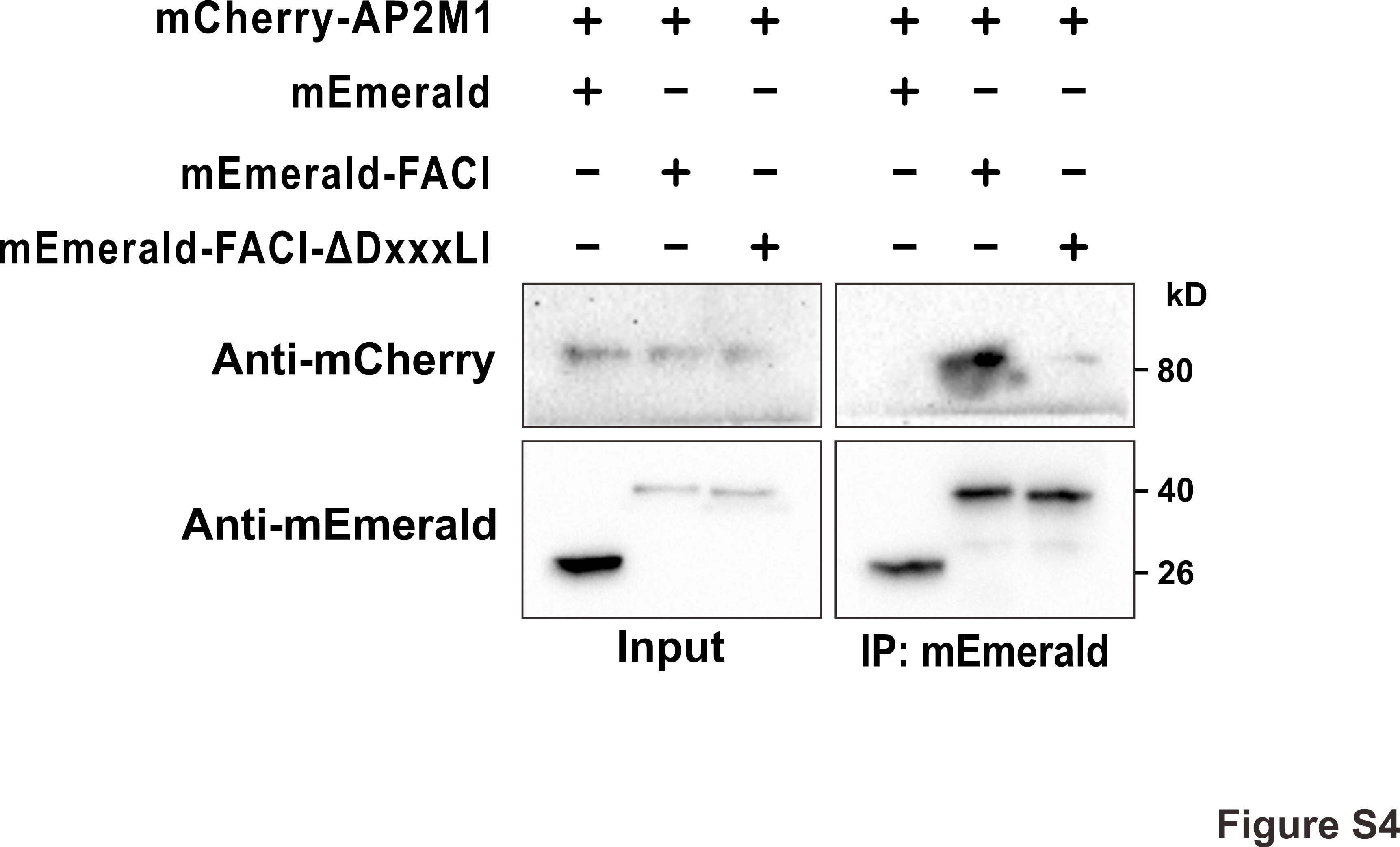

Supplement: Supplementary file 5 — Additional file 5: Figure S1. Generation of stable AML12 cells. AML12 cell lines stably expressing V5-FACI (AML12-V5-FACI) were generated and verified by immunofluorescence. A mock AML12 stable cell line (AML12-CTR) was generated and used as a control. Scale bar, 10 µm. Fig. S2. FACI localizes to CCPs in Caco-2 cells. TIRFM images. Caco-2 cells expressing mEmerald-FACI were transfected with mCherry-CLC plasmids. Scale bar, 5 μm. Fig. S3. Deletion of DxxxLI and YxxL motifs of FACI does not affect its localization to PM and ERC. (A) Confocal images of AML12 cells expressing mCherry-Rab11a and mEmerald-FACI mutants (FACI-ΔYxxL, FACI-ΔDxxxLI, and FACI-ΔYxxL-DxxxLI). Scale bar, 20 µm. (B) Confocal images of AML12 cells expressing mCherry-Rab11a and mEmerald-FACI mutants (FACI-Δ2-68 and FACI-Δ2-82). Scale bar, 10 µm. Fig. S4. AML12 cells expressing mCherry-AP2M1 were transfected with mEmerald-FACI or mEmerald-FACI-ΔDxxxLI plasmid. Cells were lysed and immunoprecipitated with anti-mCherry. Immunoprecipitates were analyzed by SDS-PAGE and probed with the indicated antibodies. Fig. S5. (A,B) AML12-mRuby2-FACI stable cells were incubated with Pitstop-2 (A), NDZ (B, upper panel) or CytD (B, lower panel) for the indicated time periods. Cells were kept on the TOKAI HIT stage-top incubator with 5% CO2 at 37°C of the microscope and imaged by SDCM at the indicated time points. Scale bar, 10 µm. (C) Quantification of the intracellular mRuby2-FACI fluorescence intensity relative to the intensity of the whole cell before (Ctrl 0 min) and after drug treatment (NDZ 90 min, CytD 90 min or Pitstop-2 75 min). n = 25-35. Statistical significance was evaluated by one-way ANOVA with Tukey's post hoc tests. Fig. S6. A C57BL/6J mice (8 weeks old, n=6) and FACI-/- mice (8 weeks old, n=7) were fed with a high-cholesterol diet for 4 weeks. Body weights, liver weights, liver cholesterol and liver triglyceride contents of mice were measured. B C57BL/6J mice (6 weeks old) were injected with AAV-FACI [file 13578_2023_1023_MOESM5_ESM.zip › supplementary images/FigS4-12Mar.tif]

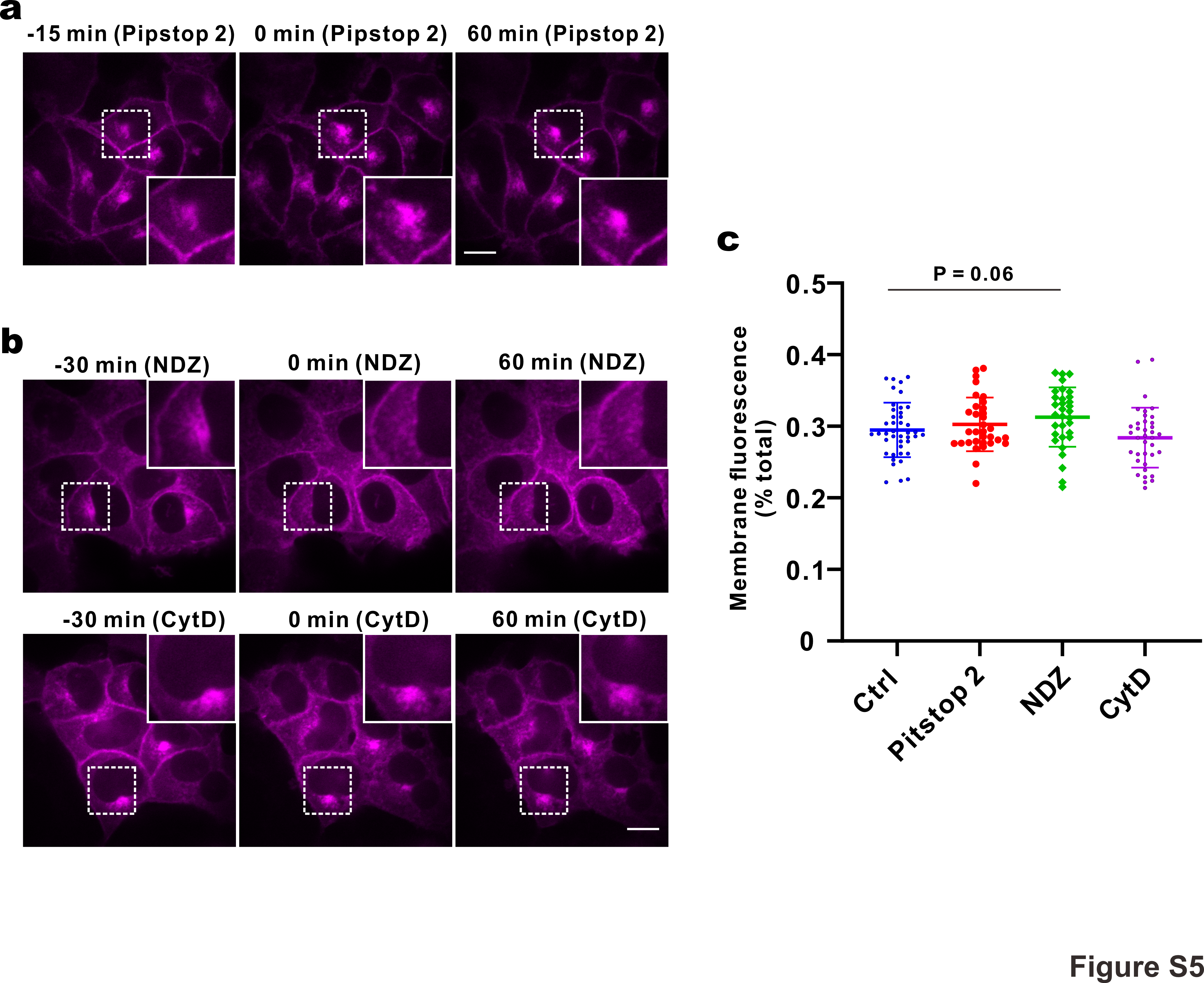

Supplement: Supplementary file 5 — Additional file 5: Figure S1. Generation of stable AML12 cells. AML12 cell lines stably expressing V5-FACI (AML12-V5-FACI) were generated and verified by immunofluorescence. A mock AML12 stable cell line (AML12-CTR) was generated and used as a control. Scale bar, 10 µm. Fig. S2. FACI localizes to CCPs in Caco-2 cells. TIRFM images. Caco-2 cells expressing mEmerald-FACI were transfected with mCherry-CLC plasmids. Scale bar, 5 μm. Fig. S3. Deletion of DxxxLI and YxxL motifs of FACI does not affect its localization to PM and ERC. (A) Confocal images of AML12 cells expressing mCherry-Rab11a and mEmerald-FACI mutants (FACI-ΔYxxL, FACI-ΔDxxxLI, and FACI-ΔYxxL-DxxxLI). Scale bar, 20 µm. (B) Confocal images of AML12 cells expressing mCherry-Rab11a and mEmerald-FACI mutants (FACI-Δ2-68 and FACI-Δ2-82). Scale bar, 10 µm. Fig. S4. AML12 cells expressing mCherry-AP2M1 were transfected with mEmerald-FACI or mEmerald-FACI-ΔDxxxLI plasmid. Cells were lysed and immunoprecipitated with anti-mCherry. Immunoprecipitates were analyzed by SDS-PAGE and probed with the indicated antibodies. Fig. S5. (A,B) AML12-mRuby2-FACI stable cells were incubated with Pitstop-2 (A), NDZ (B, upper panel) or CytD (B, lower panel) for the indicated time periods. Cells were kept on the TOKAI HIT stage-top incubator with 5% CO2 at 37°C of the microscope and imaged by SDCM at the indicated time points. Scale bar, 10 µm. (C) Quantification of the intracellular mRuby2-FACI fluorescence intensity relative to the intensity of the whole cell before (Ctrl 0 min) and after drug treatment (NDZ 90 min, CytD 90 min or Pitstop-2 75 min). n = 25-35. Statistical significance was evaluated by one-way ANOVA with Tukey's post hoc tests. Fig. S6. A C57BL/6J mice (8 weeks old, n=6) and FACI-/- mice (8 weeks old, n=7) were fed with a high-cholesterol diet for 4 weeks. Body weights, liver weights, liver cholesterol and liver triglyceride contents of mice were measured. B C57BL/6J mice (6 weeks old) were injected with AAV-FACI [file 13578_2023_1023_MOESM5_ESM.zip › supplementary images/FigS5-12Mar.tif]

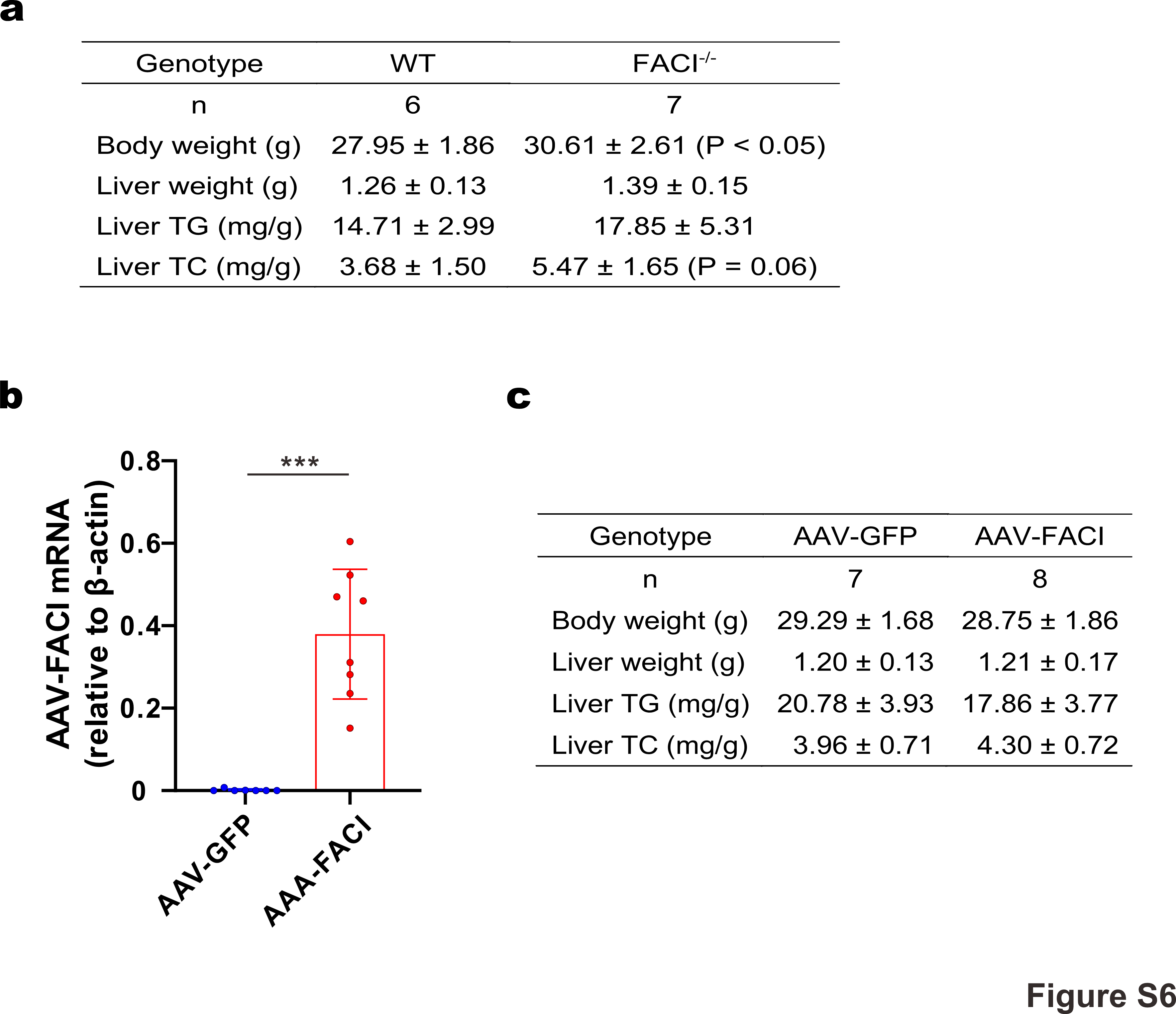

Supplement: Supplementary file 5 — Additional file 5: Figure S1. Generation of stable AML12 cells. AML12 cell lines stably expressing V5-FACI (AML12-V5-FACI) were generated and verified by immunofluorescence. A mock AML12 stable cell line (AML12-CTR) was generated and used as a control. Scale bar, 10 µm. Fig. S2. FACI localizes to CCPs in Caco-2 cells. TIRFM images. Caco-2 cells expressing mEmerald-FACI were transfected with mCherry-CLC plasmids. Scale bar, 5 μm. Fig. S3. Deletion of DxxxLI and YxxL motifs of FACI does not affect its localization to PM and ERC. (A) Confocal images of AML12 cells expressing mCherry-Rab11a and mEmerald-FACI mutants (FACI-ΔYxxL, FACI-ΔDxxxLI, and FACI-ΔYxxL-DxxxLI). Scale bar, 20 µm. (B) Confocal images of AML12 cells expressing mCherry-Rab11a and mEmerald-FACI mutants (FACI-Δ2-68 and FACI-Δ2-82). Scale bar, 10 µm. Fig. S4. AML12 cells expressing mCherry-AP2M1 were transfected with mEmerald-FACI or mEmerald-FACI-ΔDxxxLI plasmid. Cells were lysed and immunoprecipitated with anti-mCherry. Immunoprecipitates were analyzed by SDS-PAGE and probed with the indicated antibodies. Fig. S5. (A,B) AML12-mRuby2-FACI stable cells were incubated with Pitstop-2 (A), NDZ (B, upper panel) or CytD (B, lower panel) for the indicated time periods. Cells were kept on the TOKAI HIT stage-top incubator with 5% CO2 at 37°C of the microscope and imaged by SDCM at the indicated time points. Scale bar, 10 µm. (C) Quantification of the intracellular mRuby2-FACI fluorescence intensity relative to the intensity of the whole cell before (Ctrl 0 min) and after drug treatment (NDZ 90 min, CytD 90 min or Pitstop-2 75 min). n = 25-35. Statistical significance was evaluated by one-way ANOVA with Tukey's post hoc tests. Fig. S6. A C57BL/6J mice (8 weeks old, n=6) and FACI-/- mice (8 weeks old, n=7) were fed with a high-cholesterol diet for 4 weeks. Body weights, liver weights, liver cholesterol and liver triglyceride contents of mice were measured. B C57BL/6J mice (6 weeks old) were injected with AAV-FACI [file 13578_2023_1023_MOESM5_ESM.zip › supplementary images/FigS6-18Apr_ESM.tif]
